# Nucleation of α-pinene oxidation products with sulfuric acid

**DOI:** 10.1039/d6ea00046k

**Published:** 2026-06-09

**Authors:** Eva Sommer, João Almeida, Wenjuan Yu, Mario Simon, Felix Möller, Christos Xenofontos, Andrea Pozzer, Zhensen Zheng, Nirvan Bhattacharyya, Dina Alfaouri, Samira Atabakhsh, Rima Baalbaki, Hannah Beckmann, Moritz Berntheusel, Pia Bogert, Mattia Busato, Manjula Canagaratna, Lucía Caudillo-Plath, Anouck Chassaing, Romulo Cruz-Simbron, Lubna Dada, Jenna DeVivo, Jonathan Duplissy, Hamish Gordon, Manuel Granzin, Lena Große Schute, Herbert G. Hartl, Ella Hirvensalo, Aenne Jacobshagen, Bernhard Judmaier, Milin Kaniyodical Sebastian, Hannah Klebach, Paap Koemets, Matthias Kohl, Ruth Konrat, Timm Krüger, Felix Kunkler, Andreas Kürten, Markus Leiminger, Clara J. Lietzke, Lu Liu, Roy Mauldin, Bernhard Mentler, Aleksandra Morawiec, Markus Müller, Tuukka Petäjä, Alessia Pignatelli, Pedro Rato, Tobias Reinecke, Sarah Richter, Birte Rörup, Samuel Ruhl, Douglas M. Russell, Wiebke Scholz, Jiali Shen, Alexandria Stinchfield, Roseline C. Thakur, Yandong Tong, Jens Top, Nsikanabasi Silas Umo, Jakob Weissbacher, Boxing Yang, Marcel Zauner-Wieczorek, Jiangy Zhang, Antonio Amorim, Urs Baltensberger, Theodoros Christoudias, Joachim Curtius, Neil M. Donahue, Imad El Haddad, Richard C. Flagan, Armin Hansel, Hartwig Harder, Xu-Cheng He, Heikki Junninen, Markku Kulmala, Katrianne Lehtipalo, Jos Lelieveld, Ottmar Möhler, Siegfried Schobesberger, António Tomé, Rainer Volkamer, Douglas R. Worsnop, Antti Onnela, Paul M. Winkler, Jasper Kirkby

**Affiliations:** a CERN, European Organization for Nuclear Research 1211 Geneva Switzerland eva.sommer@cern.ch jasper.kirkby@cern.ch; b Faculty of Physics, University of Vienna Strudlhofgasse 4 1090 Vienna Austria; c LIP - Laboratory for Instrumentation and Experimental Particle Physics Av. Prof. Gama Pinto, n.2 1649-003 Lisboa Portugal; d Institute for Atmospheric and Earth System Research/Physics, Faculty of Science, University of Helsinki 00014 Helsinki Finland; e Goethe University Frankfurt, Institute for Atmospheric and Enviromental Sciences Altenhoeferallee 1 60438 Frankfurt am Main Germany; f Institute of Physics, Faculty of Science and Technology, University of Tartu Tartu 50411 Estonia; g Climate and Atmosphere Research Center, The Cyprus Institute 1645 Nicosia Cyprus; h Atmospheric Chemistry Department, Max Planck Institute for Chemistry Hahn-Meitner-Weg 1 55128 Mainz Germany; i Institute for Ion and Applied Physics, University of Innsbruck Technikerstraße 25 6020 Innsbruck Austria; j IONICON Analytik GmbH 6020 Innsbruck Austria; k Leibniz-Institute for Tropospheric Research Permoser Straße 15 04318 Leipzig Germany; l Institute of Meteorology and Climate Research, Karlsruhe Institute of Technology Karlsruhe Germany; m Aerodyne Research Billerica MA 01821 USA; n Department of Environmental Science, Stockholm University Stockholm 10961 Sweden; o Bolin Centre for Climate Research, Stockholm University Stockholm 10961 Sweden; p Department of Chemistry, University of Colorado Boulder Cristol Chemistry, 215 UCB Boulder CO USA; q CIRES, Cooperative Institute for Research in Environmental Sciences, University of Colorado Boulder Boulder CO 80309-0215 USA; r PSI Center for Energy and Environmental Sciences, Paul Scherrer Institute 5232 Villigen PSI Switzerland; s Department of Chemistry, Carnegie Mellon University 5000 Forbes Ave Pittsburgh PA 15213 USA; t Center for Atmospheric Particulate Studies, Carnegie Mellon University 5000 Forbes Ave Pittsburgh PA 15213 USA; u Helsinki Institute of Physics, University of Helsinki Helsinki Finland; v Department of Chemical Engineering and Center for Atmospheric Particle Studies, Carnegie Mellon University Pittsburgh PA 15213 USA; w Airmodus Oy. Erik Palmenin aukio 1 00560 Helsinki Finland; x Airel Ltd Observatooriumi 5 61602 Tõravere Estonia; y Department of Atmospheric and Oceanic Sciences, University of Colorado, Boulder Boulder CO 80309 USA; z Department of Chemistry and Biochemistry, University of North Carolina Wilmington NC 28403 USA; a Faculdade de Ciencias da Universidade de Lisboa Edificio C8 Campo Grande 1749-016 Lisboa Portugal; b Department of Engineering and Public Policy, Carnegie Mellon University 5000 Forbes Ave Pittsburgh PA 15213 USA; c California Institute of Technology Pasadena CA 91125 USA; d College of Environmental Sciences and Engineering, Peking University Beijing 100871 China; e Laboratory of Atmospheric and Environmental Sciences, University of Tartu W. Ostwaldi tn 1 Tartu 50411 Estonia; f Finnish Meteorological Institute, Helsinki Finland; g Department of Technical Physics, University of Eastern Finland PO Box 1627 70211 Kuopio Finland; h IDL-Universidade da Beira Interior Rua Marquês D’Ávila e Bolama 6201-001 Covilhã Portugal

## Abstract

Particle nucleation from trace atmospheric vapours is important for climate since it gives rise to more than half of global cloud condensation nuclei. Sulfuric acid (H_2_SO_4_) has long been recognised to drive particle nucleation in the atmosphere and, more recently, highly oxygenated products of biogenic vapours—in particular monoterpenes such as α-pinene (C_10_H_16_)—have also been shown to nucleate under atmospheric conditions, without requiring additional vapours. This raises the question of whether a nucleation synergy exists between α-pinene oxygenated organic molecules (AP-OOM) and H_2_SO_4_, as has been suggested by early studies. Here we report new particle formation from AP-OOM and H_2_SO_4_ in the absence of base vapours such as ammonia (NH_3_), measured in experiments performed with the CERN CLOUD (Cosmics Leaving Outdoor Droplets) chamber at cool boundary layer temperatures of −10 °C and +5 °C. We find that AP-OOM nucleation rates increase strongly when H_2_SO_4_ concentrations exceed around 10^6^ cm^−3^. The enhancement is synergistic and cannot be explained as a simple linear addition of independent chemical systems. Above this threshold, the nucleation rate depends approximately linearly on H_2_SO_4_ concentration, in contrast with the strong sensitivity to H_2_SO_4_ for H_2_SO_4_–NH_3_ nucleation. Nucleation rates are 10–100-fold higher in the presence of ions from galactic cosmic rays or from the CERN pion beam. Based on these measurements, we have parameterised a temperature-dependent H_2_SO_4_-AP-OOM nucleation rate in the absence of base vapours and implemented it in the EMAC (ECHAM/MESSy Atmospheric Chemistry) Earth system model. In comparison with a parameterisation developed in an earlier study [Riccobono *et al.*, *Science*, 2014, **344**, 717–721.], the new parameterisation indicates sharply reduced nucleation rates in the boundary layer over warm regions, and increased rates over northern boreal forests.

## Abstract 2

A new parametrisation of synergistic nucleation from alpha-pinene oxidation products and sulfuric acid in the absence of base vapours.

## Graphical abstract

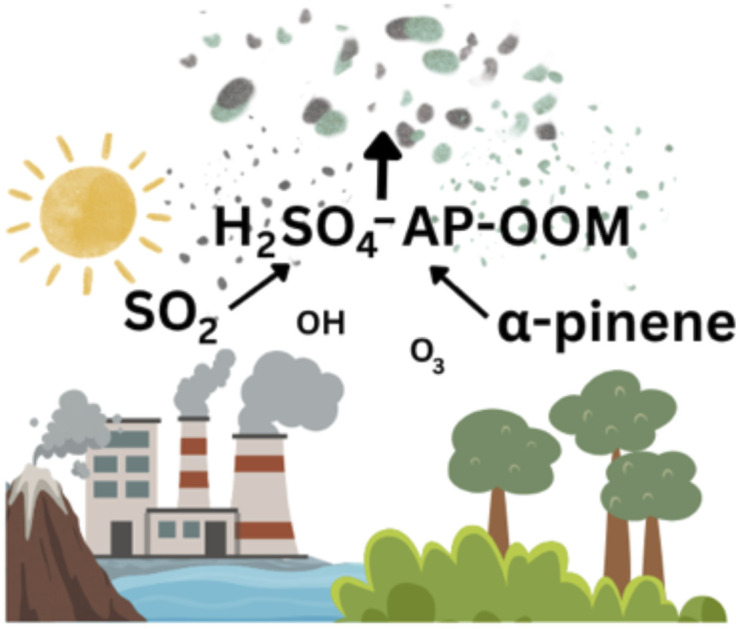

**Environmental significance**

Accurate representation of new particle formation from sulfuric acid and from oxidized organics is crucial for capturing aerosol effects in global climate models. Existing parameterisations are based on limited experimental conditions and do not accurately capture combined acid/organic nucleation effects or their temperature dependence. Here, we report chamber measurements of nucleation rates from α-pinene oxidation products and sulfuric acid in the near-absence of base vapours such as ammonia, under conditions representative of the cool boundary layer. We show that their synergistic interaction enhances particle formation beyond either component alone. Based on these results, we develop a parameterisation accounting for vapour concentrations, ionisation and temperature. This framework improves the representation of a major aerosol formation source in global models.

## 1 Introduction

Aerosol particles and aerosol–cloud interactions are the largest source of uncertainty in global climate projections.^1^ It is estimated that more than half of all cloud condensation nuclei (CCN) arise from new particle formation (NPF).^2^ NPF involves the formation of molecular clusters from low-volatility vapours, a nucleation process. Once these clusters exceed a diameter of 1–2 nm they become stable against evaporation and, if not lost to pre-existing aerosol particles or other surfaces, may grow above sizes of around 50 nm where they can act as CCN.^3^

Sulfuric acid (H_2_SO_4_) has long been recognised as a key driver of atmospheric new particle formation across a wide range of environments.^4–6^*Via* autoxidation,^7^ biogenic vapours such as α-pinene (C_10_H_16_) can also rapidly give rise to highly oxygenated compounds with extremely low volatility. These compounds can condense onto particles and drive their growth, forming secondary organic aerosol and CCN over extensive continental regions.^8^ Highly oxygenated organic molecules from α-pinene (AP-OOM) have been shown to nucleate even in the absence of H_2_SO_4_.^9^ Alpha-pinene is the most abundant monoterpene, and its low-volatility oxidation products play an important role in global CCN production, especially in the pristine pre-industrial atmosphere.^2^ While isoprene (C_5_H_8_) suppresses particle formation in the boundary layer,^10^ a recent study at the CERN CLOUD (Cosmics Leaving OUtdoor Droplets) chamber showed that isoprene oxygenated organic molecules (IP-OOM) can drive NPF at the cold temperatures of the upper-troposphere.^11^ The nucleation rates from IP-OOM were enhanced by about two orders of magnitude in the presence of trace amounts of H_2_SO_4_.^11^ This raises the question of whether a nucleation synergy also exists between AP-OOM and H_2_SO_4_ in the absence of base vapours, as suggested by previous experiments.^12–14^

Global climate models require parameterisations of nucleation rates in order to evaluate the climate impact of NPF and CCN.^15^ A temperature- and ionisation rate-dependent parameterisation of H_2_SO_4_(–NH_3_)–H_2_O nucleation was developed from experiments at the CERN CLOUD chamber by Dunne *et al.*^6^ and has been implemented in several global climate models. Kirkby *et al.*^9^ parameterised pure AP-OOM nucleation rates at +5 °C. The mixed system of H_2_SO_4_ with oxygenated organics and no added base vapours was investigated at CLOUD by Riccobono *et al.*,^12^ while Lehtipalo *et al.*^14^ investigated the system of H_2_SO_4_ and oxygenated organics with different levels of NO_*x*_ and NH_3_ at +5 °C.

Riccobono *et al.*^12^ used a quantity referred to as BioOxOrg as a proxy for α-pinene oxygenated organic molecules. The BioOxOrg was produced from pinanediol (PD; C_10_H_18_O_2_), a first-generation oxidation product of α-pinene. BioOxOrg was defined as the total reaction products from PD oxidation by hydroxyl (OH) radicals, so it included compounds with a wide range of oxygen content. A subset of BioOxOrg comprised the same highly-oxygenated AP-OOM compounds measured in the present study that drive nucleation.

Riccobono *et al.*^12^ made an important advance at the time by revealing rapid formation of highly-oxygenated α-pinene products with sufficiently low volatility that, together with H_2_SO_4_, could account for nucleation rates observed in the atmosphere. However, the experiments were limited in several respects: pure biogenic nucleation was unknown, and no experiments were performed with H_2_SO_4_ below 10^6^ cm^−3^; a pinanediol proxy was used instead of α-pinene so the BioOxOrg did not include the high yield of extremely low volatility OOM from ozone-initiated autoxidation of α-pinene; contaminant ammonia, which strongly affects nucleation rates from H_2_SO_4_, could not be excluded below around 30 pptv; and the experiments were carried out at a single temperature (5 °C). The resulting parameterisation of the nucleation rate is a simple power law, *J* = *k*[H_2_SO_4_]^*p*^[BioOxOrg]^*q*^, with no dependence on ionisation rates or temperature.^12^

Here we address these limitations in experiments performed at the CERN CLOUD chamber^4^ to investigate nucleation of AP-OOM with trace concentrations of H_2_SO_4_ and in the absence of base vapours (<2 pptv NH_3_ contaminant level). We report nucleation rates from AP-OOM measured under cool boundary layer conditions between −10 °C and +5 °C, both in the presence and absence of H_2_SO_4_. We also quantify the contribution of ion-induced nucleation. The measurements allow us to characterise the synergy between AP-OOM and H_2_SO_4_ and to parameterise the nucleation rate as a function of vapour concentrations, ionisation rate and temperature.

## 2 Methods

### 2.1 The CERN CLOUD experiment

We conducted all measurements at the CERN CLOUD experiment.^4^ The CLOUD chamber is a 26 m^3^ continuously-stirred stainless-steel reactor. The chamber has ultra-low contaminants, so controlled experiments can be performed with trace gases at atmospheric concentrations. New particle formation experiments are performed under steady-state conditions, where the loss rates of gases and particles balance their injection or production rates. Trace gas concentrations, particle size distributions, and chamber conditions are monitored using a suite of state-of-the-art instruments that continuously sample the contents of the chamber. A continual injection of around 380 L min^−1^ humidified ultrapure air plus precursor vapours compensates for the sampling losses.

### 2.2 Chamber conditions

We conducted the experiments during the CLOUD15, CLOUD16, and CLOUD17 campaigns in autumn 2022, 2023, and 2024, respectively. We investigated new particle formation from oxygenated organic molecules derived from α-pinene and H_2_SO_4_ under conditions representative of the cool boundary layer. Experiments were performed at temperatures of −10 °C and +5 °C.

We injected α-pinene into the chamber from an evaporator and oxidised it *via* ozonolysis to form AP-OOM. H_2_SO_4_ was produced *in situ* by oxidation of SO_2_ with OH radicals generated from photolysis of O_3_ with ultraviolet radiation below 320 nm.^16^

We measured nucleation rates for AP-OOM concentrations ranging from <10^5^ cm^−3^ (instrument detection limit) to 10^8^ cm^−3^, and for H_2_SO_4_ concentrations between <10^4^ cm^−3^ (instrument detection limit) and approximately 10^7^ cm^−3^.

At selected vapour concentrations, we measured nucleation rates under three ionisation conditions: (a) neutral conditions, where all ions were removed from the chamber using an electric clearing field, (b) GCR conditions, where the chamber was exposed to ground level ionisation from galactic cosmic rays, and (c) beam conditions, where elevated ionisation rates were generated using a π^+^ beam from the CERN Proton Synchrotron, simulating ionisation at higher altitudes. These three ionisation conditions are referred to as neutral, GCR, and beam, respectively.

We maintained the relative humidity at ∼60% at both temperatures. Ozone (O_3_) concentrations were ∼80 ppbv during the −10 °C experiments and ∼40 ppbv during the +5 °C experiments. All experiments in CLOUD16 and CLOUD17, except for one reference run per campaign, were conducted in the presence of UV illumination.^16^ No NH_3_ was added to the chamber during or immediately before the experiments. The chamber background NH_3_ concentration is estimated to be below 2 pptv, based on APi-TOF^17^ measurements showing a very low abundance of NH_3_-containing H_2_SO_4_ clusters under comparable conditions.^18^ The CERN CLOUD chamber is operated as a continuously stirred steady-state reactor with a constant flow. The total air flow in our experiments was 370-380 L min^−1^, corresponding to a trace-gas residence time of ∼70 min. Two mixing fans mounted at the top and bottom of the chamber ensured uniform trace gas distributions. All nucleation and growth rates reported here were obtained under steady-state conditions. We studied the mixed H_2_SO_4_-AP-OOM system during the CLOUD16 and CLOUD17 campaigns and the pure biogenic system during CLOUD15. For the development of the H_2_SO_4_-AP-OOM parameterisation, we additionally include nucleation rates for the H_2_SO_4_-AP-OOM system reported previously by Kirkby *et al.*^9^

### 2.3 Precursor gas and condensable vapour measurement

We measured α-pinene concentrations using proton-transfer-reaction mass spectrometry (PTR-MS) during all campaigns. During CLOUD15 and CLOUD17, α-pinene was measured with a H_3_O^+^-PTR-MS (STOF).^19,20^ The lower limit of detection of the STOF instrument for α-pinene is ∼150 pptv. During CLOUD16, a FUSION PTR-MS,^21^ with a sensitivity for α-pinene down to < 1 pptv, was used as the primary α-pinene measurement, while the STOF was measuring in parallel to allow an instrument comparison. Both instruments were regularly calibrated using gas standards, and instrumental backgrounds were subtracted. We used the measured α-pinene concentrations from both instruments to develop an instrument- and campaign-independent relationship allowing us to calculate α-pinene concentrations in the chamber directly from the injection rate (*i.e.* mass flow controller, MFC, settings). A more detailed description of this approach is provided in the SI.

H_2_SO_4_ and AP-OOM were measured using a nitrate chemical ionisation atmospheric pressure interface time-of-flight mass spectrometer (NO_R_^−^-CI-APi-TOF, hereafter referred to as nitrate-CIMS).^22,23^ H_2_SO_4_ concentrations were calibrated in each campaign using a well-established procedure,^24,25^ yielding absolute concentrations. We assume the same detection efficiency for AP-OOM as for H_2_SO_4_. This is appropriate for the more highly oxygenated molecules (HOM)^26^ with O ≥ 6, which are the subset of AP-OOM that drive NPF. The overall uncertainty of nitrate-CIMS measurements is a factor of two. A more detailed instrument description is provided in the SI.

### 2.4 Definition of AP-OOM

Previous CLOUD studies have shown that AP-OOM nucleation and early growth is driven by exceedingly low volatility compounds,^27–29^ which are known as extremely-low-volatility- and ultra-low-volatility organic compounds (ELVOC and ULVOC, respectively) in the framework of the Volatility Basis Set, VBS.^30,31^ For this work, AP-OOM are defined as the entire subset of oxygenated compounds from α-pinene that are measured by a nitrate-CIMS, as previously used by Wang *et al.*;^32^ no additional requirements (number of carbon atoms, O : C ratio or mass range) are applied.

Nitrate chemical ionisation is sensitive to AP-OOM with more than around 6 oxygen atoms.^33^ Since all our experiments are without NO_*x*_, the detected OOM consist exclusively of C_*x*_H_*y*_O_*z*_ compounds. In the temperature range of our study, nitrate chemical ionisation therefore efficiently detects ELVOC and ULVOC, while discriminating against less-volatile compounds.^34^ We measure a mean AP-OOM yield per ozonolysis reaction (including secondary OH reactions) of 6.0%. Within experimental differences, this is consistent with the ELVOC yields for α-pinene oxidation (3.4% from O_3_ and 0.44% from OH) reported by Jokinen *et al.*^35^ using a nitrate-CIMS, which are used in the EMAC model simulations reported here.

To ensure consistent AP-OOM concentrations across campaigns, we compared the measured nitrate-CIMS signal to the α-pinene ozonolysis rate, using the measured O_3_ concentration and the α-pinene concentration derived from MFC settings, after calibration with mass spectrometers. We established separate temperature-dependent relationships for −10 °C and +5 °C and applied them to the datasets from CLOUD15–17. For earlier data from Kirkby *et al.*,^9^ direct AP-OOM measurements were not available. We therefore estimate AP-OOM from the nitrate-CIMS C_10_ organic signal, which correlates strongly with AP-OOM in CLOUD15–17 (*R*^2^ = 0.99). Further details are provided in the SI and Fig. S1.

### 2.5 Aerosol particle and charged cluster measurement

We measured naturally charged clusters with an Aerodyne Research Inc. APi-TOF operated in negative-ion mode.^17^ Particles up to ∼3 nm were measured with an Airmodus Ltd nano-condensation nucleus counter (nCNC) consisting of a particle size magnifier (PSM)^36^ coupled to a butanol condensation particle counter (CPC). We monitored total particle number concentrations above 2.5 nm with a butanol CPC (TSI 3776). We measured particle number size distributions from 2–66 nm and 18–533 nm with two Scanning Mobility Particle Sizer (SMPS) systems:^37^ a nano-SMPS (TSI 3938) coupled to a butanol CPC (TSI 3776) and a long-SMPS (TSI 3082) coupled to a butanol CPC (TSI 3775), respectively. We used an Airel Ltd. Neutral cluster and Air Ion Spectrometer (NAIS),^38^ to measure the naturally charged particle number size distribution from ∼1 to 41 nm, as well as the total particle number size distribution from 2 to 42 nm in both positive and negative polarity.

We determined particle growth rates (GRs) from 3.2 to 8 nm using the appearance-time method described in Dada *et al.*^39^ In CLOUD15, we obtained the input size distributions for the growth-rate calculation with a DMA-train configured as described by Stolzenburg *et al.*^40^ In CLOUD16 and CLOUD17, we obtained the size distributions for the growth rate calculation from NAIS measurements.

We calculated the nucleation rates of particles at 1.7 nm diameter threshold (*J*_1.7_) following the method described in Dada *et al.*^39^ Ideally, when measuring and parameterising nucleation rates, one would choose the size threshold to correspond to the critical size, *i.e.* the size at which the growth rate of a particle begins to exceed its evaporation rate. However, the critical size depends on the chemical system and temperature. CLOUD has therefore chosen a practical solution to quote particle nucleation rates at 1.7 nm mobility diameter, which is safely above the critical size for all chemical systems that we have studied so far. For example, in the case of sulfuric acid, it corresponds around 9 H_2_SO_4_ molecules, whereas the critical cluster contains 2–4 H_2_SO_4_ molecules equivalent to 1.1–1.3 nm mobility diameter. However, with this choice, under slow GR conditions the condensation sink (CS) can reduce the survival probability of the particle in growing from the critical size to 1.7 nm. At CLOUD the CS under standard conditions is 2.2 × 10^−3^ s^−1^, equal to the wall loss rate. Our studies have shown that, for a GR equivalent to above around 3 × 10^6^ cm^−3^ H_2_SO_4_, *i.e.* 0.6 nm h^−1^, in the CLOUD chamber, the difference between *J*_1.7_ and the nucleation rate at the critical size is relatively small.^41^

### 2.6 EMAC global model implementation

Based on our measurements, as well as the inorganic H_2_SO_4_–NH_3_ parameterisation from Dunne *et al.*,^6^ we derived a new parameterisation for the mixed H_2_SO_4_-AP-OOM nucleation mechanism and implemented it in a global model. We performed all simulations with the ECHAM/MESSy Atmospheric Chemistry (EMAC) model, which integrates the ECHAM5 general circulation model^42^ with the MESSy2 framework^43^ to represent meteorology, trace gases, and aerosol processes. Further information on the model configuration, as well as a full description of the H_2_SO_4_-AP-OOM parameterisation developed in this study, is provided in the SI.

## 3 Experimental results

### 3.1 H_2_SO_4_-AP-OOM nucleation

**Fig. 1:**
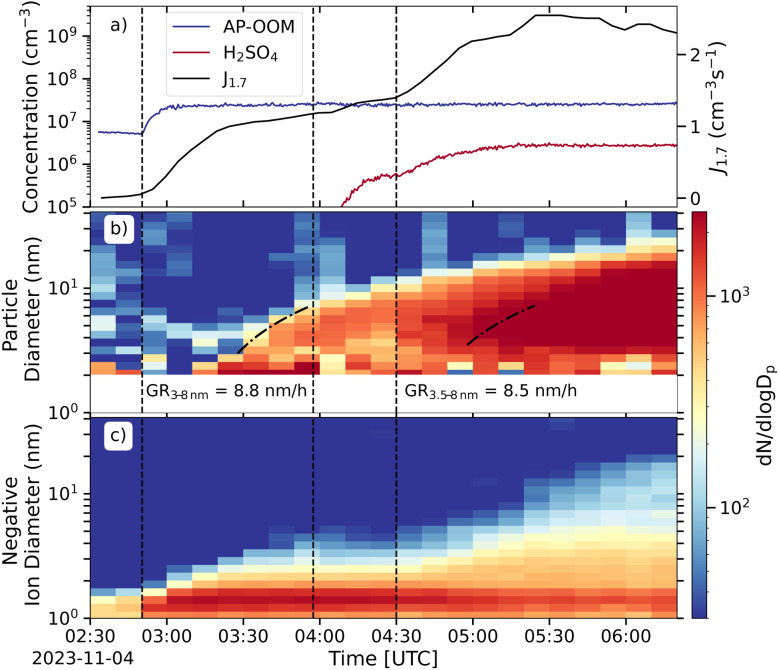
Evolution of an example nucleation experiment at −10 °C under GCR conditions. (a) AP-OOM and H_2_SO_4_ concentrations (left axis) and the corresponding nucleation rate at 1.7 nm, *J*_1.7_ (right axis). (b) Total particle number size distribution between 2 and 40 nm, measured by the NAIS. The dashed black curves show particle growth from ∼3 to 8 nm, together with their fitted growth rates. (c) Naturally-charged size distribution of negative ions and charged particles between 1 and 40 nm, measured by the NAIS. Vertical dashed lines indicate when steady-state gas concentrations were changed: first an increase of AP-OOM and then, in two steps, an increase of SO_2_ to produce H_2_SO_4_. The experimental conditions are 80 ppbv O_3_, <2 pptv NH_3_, 120 pptv α-pinene, 0–100 pptv SO_2_, 60% RH and −10 °C.

Fig. 1 illustrates a representative experiment. We first establish pure biogenic nucleation from AP-OOM and subsequently introduce SO_2_ to produce H_2_SO_4_ and quantify its effect on the nucleation rate. The experiment shown in Fig. 1 was conducted at −10 °C during the CLOUD16 campaign under galactic cosmic ray (GCR) ionisation with ionisation rate *Q* ≈ 2 cm^−3^ s^−1^, at approximately 60% relative humidity (RH), ∼80 ppbv O_3_, and continuous UV illumination.^16^ Prior to each experiment, we cleaned the chamber by operating the mixing fans at maximum rotation speed to increase the loss rates of aerosol particles and condensable vapours. During these cleaning periods, we establish stable conditions of temperature, humidity, O_3_, and α-pinene concentrations. For all experiments reported here, no NH_3_ was added to the chamber during or prior to the measurements. The low abundance of NH_3_-containing H_2_SO_4_ clusters observed in the APi-TOF is consistent with contaminant NH_3_ concentrations below 2 pptv.^18^

The first vertical dashed line in Fig. 1 indicates the start of the experiment, where we reduced the mixing fan speed from 100 to 12%. This reduces the loss rate of condensable vapours and increases their lifetime in the chamber. AP-OOM reach a new equilibrium concentration of around 3 × 10^7^ cm^−3^ (Fig. 1 panel a). The new steady-state AP-OOM concentration initiated new particle formation with particles emerging directly from the ion band (Fig. 1c), which is consistent with the strong ion-induced contribution previously observed for pure biogenic nucleation.^9^

After the AP-OOM-driven nucleation rate reached a steady state, we introduced SO_2_, which is rapidly oxidised by OH to form H_2_SO_4_. We increased the H_2_SO_4_ concentration in two steps (Fig. 1a), reaching approximately 2 × 10^6^ cm^−3^. At this relatively low H_2_SO_4_ concentration, the nucleation rate doubled from ∼1.2 to 2.5 cm^−3^ s^−1^. Taking only the H_2_SO_4_–NH_3_ nucleation mechanism from Dunne *et al.*^6^ into account, we would expect *J*_1.7_ ∼ 10^−5^ cm^−3^ s^−1^ at 2 pptv contaminant NH_3_. Even if we assume an unrealistically high NH_3_ concentration of 100 pptv, the H_2_SO_4_–NH_3_nucleation rate predicted by Dunne *et al.*^6^ would remain below 4 × 10^−4^ cm^−3^ s^−1^. We therefore attribute the observed increase in nucleation rate in Fig. 1 to a synergetic H_2_SO_4_-AP-OOM nucleation pathway rather than to an independent contribution from H_2_SO_4_–NH_3_ nucleation. Within experimental errors, the particle growth rate remained unchanged following the addition of 2 × 10^6^ cm^−3^ H_2_SO_4_ (Fig. 1b), consistent with the small expected increase (∼0.2 nm h^−1^).^44^

### 3.2 Charged cluster composition

**Fig. 2:**
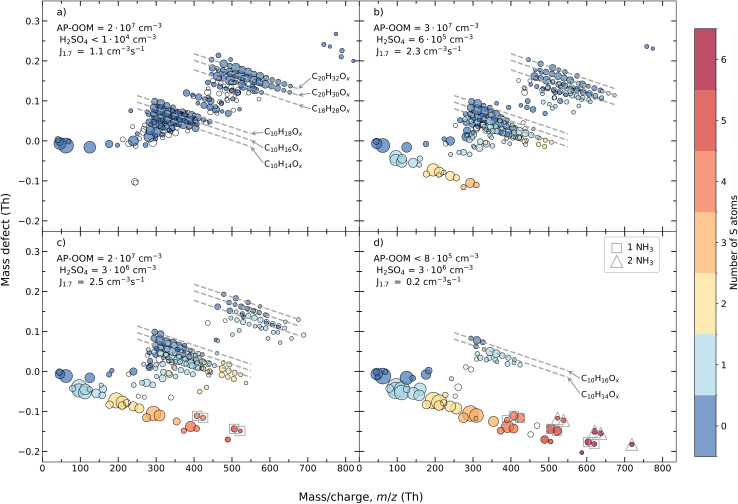
Molecular composition of negatively charged clusters during H_2_SO_4_-AP-OOM nucleation events at −10 °C. Mass defect (difference from integer mass) *versus m*/*z* during H_2_SO_4_-AP-OOM nucleation events measured with an APi-TOF in negative mode. Panels are ordered by increasing ratio of H_2_SO_4_ to AP-OOM: (a) < 0.0007, (b) 0.02, (c) 0.1, and (d) > 2. All experiments were performed with comparable levels of contaminant vapours. The symbol radius is proportional to the logarithm of the signal intensity, and the colour indicates the number of sulfur atoms in the cluster. Clusters with no sulfur atoms generally contained nitrate ions (NO_3_^−^). Square outlines indicate clusters containing one NH_3_, and triangle outlines denote clusters containing two NH_3_ molecules; no clusters were measured with more than two NH_3_ molecules. The experimental conditions are 80 ppbv O_3_, < 2 pptv NH_3_, < 10–120 pptv α-pinene, 60% RH and −10 °C.

Fig. 2 shows APi-TOF mass-defect plots of negatively charged clusters for four steady-state nucleation events at −10 °C, spanning a range of H_2_SO_4_ and AP-OOM relative concentrations. Panels (a)–(d) are ordered by increasing H_2_SO_4_ : AP-OOM ratio. In each panel, the mass defect (Th) is plotted against the nominal mass-to-charge ratio (Th), such that clusters with similar elemental composition form characteristic bands.^17,27,45^ The symbol colour indicates the number of sulfur atoms in the cluster, and the symbol radius is proportional to the logarithm of the signal intensity.

Fig. 2a represents pure biogenic AP-OOM nucleation and exhibits the characteristic C_10_ monomer (Th ≈ 240–420) and C_20_ dimer (Th ≈ 400–620) bands of AP-OOM.^27^ No sulfur-containing clusters are detected; instead, under these conditions the AP-OOM species are clustered with nitrate anions (NO_3_^−^) and AP-OOM^−^ ions. As the H_2_SO_4_ : AP-OOM ratio increases panels (b) to (d), sulfur-containing clusters become increasingly abundant. In panels (b) and (c), the contribution of HSO_4_^−^ anions appears and grows. Moreover, the organic bands include a substantial fraction containing two sulfur atoms, indicating that neutral H_2_SO_4_ is also present in the mixed clusters, and demonstrating synergistic nucleation of H_2_SO_4_-AP-OOM.

**Fig. 3:**
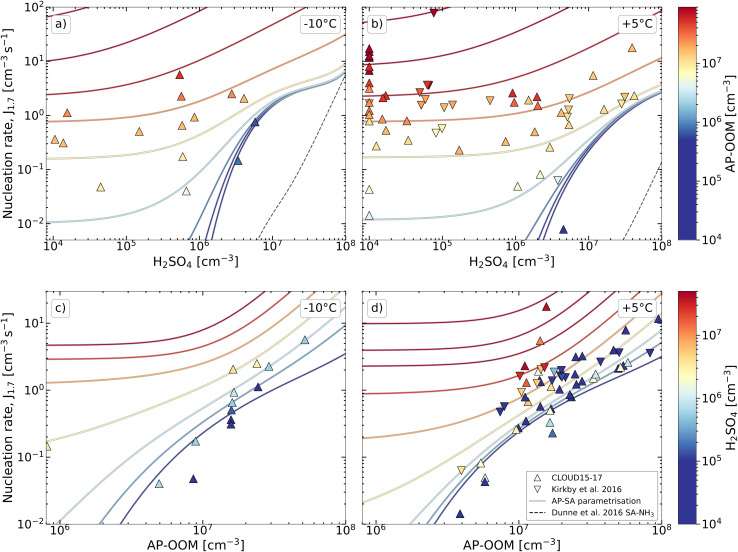
Measured and modelled nucleation rates, *J*_1.7_. The nucleation rates are shown as a function of (a) and (b) H_2_SO_4_ and (c) and (d) AP-OOM concentrations, and at −10 °C (panels a and c) and +5 °C (panels b and d). The symbols denote measured nucleation rates and the curves show those predicted by the H_2_SO_4_-AP-OOM parameterisation developed in this study. The colours indicate (a) and (b) AP-OOM, and (c) and (d) H_2_SO_4_ concentrations. In (a) and (b), AP-OOM concentrations below 10^6^ cm^−3^ are represented with a single colour due to experimental uncertainties. The inverted triangles indicate measurements from Kirkby *et al.*,^9^ while upright triangles represent measurements from the CLOUD15–17 campaigns. The black dashed lines in panels (a) and (b) indicate nucleation rates expected from H_2_SO_4_–NH_3_ (ref. 6) assuming 2 pptv contaminant NH_3_. The dark blue triangles in (a) and (b) correspond to nucleation rates measured in the presence of contaminant AP-OOM near the detection limit. All data shown here were obtained under GCR ionisation conditions with ionisation rates *Q* ≈ 2 cm^−3^ s^−1^. The experimental conditions are (a) and (c) −10 °C, 70–90 ppbv O_3_, (b) and d() +5 °C, 40–50 ppbv O_3_, and (a–d) <2 pptv NH_3_, 0–4000 pptv α-pinene and 60% RH.

Clusters with negative mass defect correspond to H_2_SO_4_ and H_2_SO_4_–NH_3_ clusters. NH_3_-containing clusters are not observed until the H_2_SO_4_ : AP-OOM ratio exceeds ∼0.1 (Fig. 2c). The average number of NH_3_ molecules per cluster remains well below two, even in the most sulfur-rich cases. This holds true even for the largest clusters containing up to seven H_2_SO_4_ molecules. This is consistent with chamber NH_3_ contamination below ∼2 pptv.^18^ In the H_2_SO_4_-dominated experiment (panel d), some organic contaminants are visible near the nitrate-CIMS detection limit for AP-OOM (∼1 × 10^5^ cm^−3^). These background organics account for the high nucleation rates observed under the lowest AP-OOM conditions shown in Fig. 3.

### 3.3 Nucleation rates *versus* H_2_SO_4_ and AP-OOM

Fig. 3 shows nucleation rates at 1.7 nm, *J*_1.7_, measured during CLOUD15–17 as well as those reported in Kirkby *et al.*^9^ Panels (a) and (b) show *J*_1.7_ as a function of H_2_SO_4_ coloured by AP-OOM concentrations at −10 °C and +5 °C, respectively. Panels (c) and (d) show the same data, but with AP-OOM on the horizontal axis, coloured by H_2_SO_4_ concentration. For these AP-OOM concentrations, the particle growth rates of all experiments exceed around 2 nm h^−1^ (see Fig. S2 in the SI) so the *J*_1.7_ measurements closely represent the nucleation rate at the critical size. The solid lines indicate *J*_1.7_ predicted by the H_2_SO_4_-AP-OOM parameterisation developed in this study. All nucleation rates shown in Fig. 3 were obtained under GCR conditions with ionisation rates of *Q* ≈ 2 cm^−3^ s^−1^ (additional data under neutral or beam conditions are not shown for clarity). In panels (a) and (b), data points without added H_2_SO_4_ are plotted at 10^4^ cm^−3^, corresponding to the instrumental lower limit of detection for H_2_SO_4_. The actual H_2_SO_4_ concentrations were likely to be substantially lower. The dashed lines in panels (a) and (b) indicate the nucleation rates expected for H_2_SO_4_–NH_3_, calculated according to Dunne *et al.*^6^

Panels (a) and (b) show, at both temperatures, that nucleation rates at constant AP-OOM are insensitive to increasing H_2_SO_4_ below a certain H_2_SO_4_ concentration threshold, spanning the region where nucleation is purely from AP-OOM. This region extends up to H_2_SO_4_ concentrations of approximately 10^5^ cm^−3^ at −10 °C and 10^6^ cm^−3^ at +5 °C. Below these thresholds, H_2_SO_4_ has no measurable effect on *J*_1.7_, but once these thresholds are exceeded, the addition of H_2_SO_4_ leads to a pronounced increase in nucleation rates. For example, at −10 °C, increasing H_2_SO_4_ to ∼3 × 10^6^ cm^−3^ in the presence of ∼1.5 × 10^7^ cm^−3^ AP-OOM enhances *J*_1.7_ by a factor of approximately seven, while the same increase at +5 °C raises *J*_1.7_ by a factor of about five. These changes of H_2_SO_4_ would result in nucleation rates around 5 orders-of-magnitude lower for H_2_SO_4_–NH_3_.^6^ We therefore attribute the observed enhancement to synergetic H_2_SO_4_-AP-OOM nucleation. The near-linear dependency of *J*_1.7_ on acid concentration above the threshold is similar to the findings of Shen & Russell *et al.*^11^ for H_2_SO_4_-IP-OOM, but the acid enhancement is stronger for IP-OOM. A similar near-linear dependency of *J*_1.7_ on AP-OOM is seen above the threshold for H_2_SO_4_-AP-OOM nucleation (Fig. 3c and d).

We also observe that extremely low concentrations of AP-OOM have a strong effect on nucleation rates from the inorganic system. The dark blue triangles in Fig. 3a and b show measurements of nucleation rates with only contaminant levels of AP-OOM in the chamber, close to the detection limit of the nitrate-CIMS. Under these conditions, organic vapours alone do not produce measurable nucleation rates, yet the observed rates substantially exceed those predicted for purely inorganic H_2_SO_4_–NH_3_ nucleation, indicated by the dashed lines.^6^ This demonstrates that even very low concentrations of AP-OOM efficiently activate a mixed H_2_SO_4_-AP-OOM nucleation pathway, leading to a large increase in *J*_1.7_, despite organic vapours being insufficient to drive nucleation on their own.

### 3.4 Nucleation rates *versus* ion concentrations

**Fig. 4:**
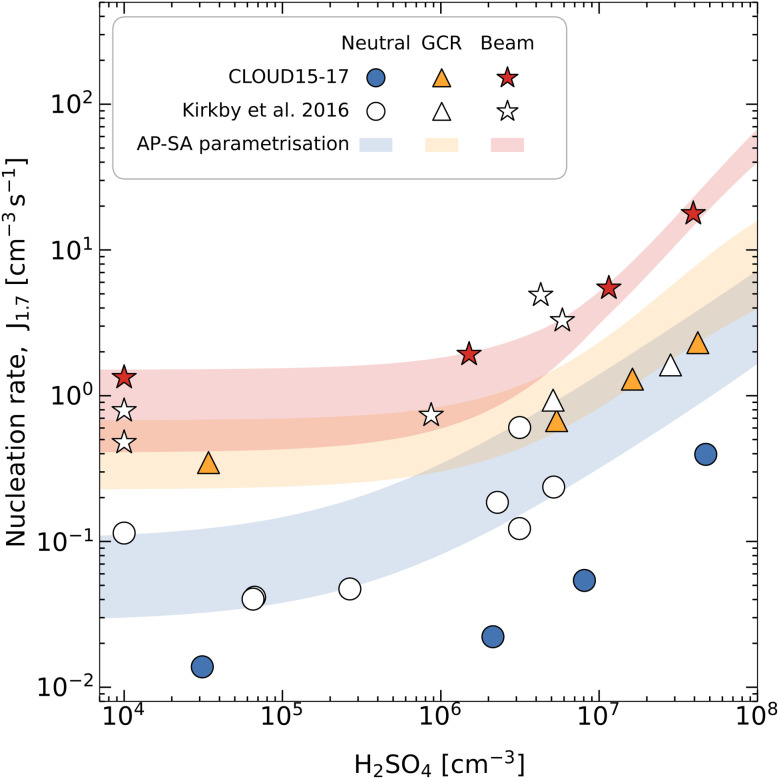
Nucleation rates under different ionisation conditions at +5 °C. The symbols show measured nucleation rates *versus* SA at fixed AP-OOM of (1.0–1.3) × 10^7^ cm^−3^. The coloured bands indicate the modelled nucleation rates based on the parameterisation developed in this study, and their width reflects the range of AP-OOM and ion concentrations in the experimental data. The circles and blue shading correspond to neutral conditions (ion pair production rate, *Q* = 0 cm^−3^ s^−1^), the triangles and orange shading correspond to GCR conditions (*Q* = 1.8–12 ion pairs cm^−3^ s^−1^, depending on the presence of background muons from the CERN Proton Synchrotron), and the stars and red shading correspond to pion beam conditions (50–100 ion pairs cm^−3^ s^−1^). The hollow symbols denote measurements from Kirkby *et al.*,^9^ and coloured symbols are measurements from the CLOUD15–17 campaigns. The experimental conditions are 40–50 ppbv O_3_, <2 pptv contaminant NH_3_, 60% RH and +5 °C.

Fig. 4 shows nucleation rates *J*_1.7_ measured at +5 °C with AP-OOM concentrations of 1.0–1.3 × 10^7^ cm^−3^ over a range of H_2_SO_4_ concentrations and ionisation conditions, together with the corresponding rates predicted by the H_2_SO_4_-AP-OOM parameterisation (see Section 3.5), represented by the coloured bands. Neutral conditions (*Q* = 0 cm^−3^ s^−1^) are shown as circles, galactic cosmic ray (GCR) background ionisation as triangles, and pion-beam conditions as stars. Experiments without added H_2_SO_4_ are set to 10^4^ cm^−3^, corresponding to the instrumental lower limit of detection for H_2_SO_4_.

Fig. 4 shows a strong ion-induced enhancement of *J*_1.7_ for both AP-OOM and H_2_SO_4_-AP-OOM and for all ionsation rates from 2 to 100 ion pairs cm^−3^ s^−1^. Moreover, for all ion conditions, the addition of H_2_SO_4_ above ∼10^6^ cm^−3^ leads to a substantial increase in nucleation rates relative to pure AP-OOM nucleation. This demonstrates that the H_2_SO_4_-AP-OOM synergy is present for both charged (ion-induced) and neutral molecular clusters. Under neutral conditions, the addition of ∼5 × 10^7^ cm^−3^ H_2_SO_4_ increases *J*_1.7_ by more than a factor of 20, which is comparable to the enhancement of GCR compared with neutral conditions.

In Fig. 4 the neutral nucleation rates measured during CLOUD15–17 are systematically lower than those reported by Kirkby *et al.*^9^ The neutral channel is especially sensitive to small changes in background impurities in the chamber, which can cause systematic discrepancies between campaigns. Our parameterisation includes all data in the fitted values and, despite these systematic uncertainties, reproduces the measured nucleation rates well across the full range of conditions (see Section 4).

## 4 Parameterisation of nucleation rates

We have developed a temperature-dependent parameterisation of H_2_SO_4_-AP-OOM nucleation which follows a similar approach as that for H_2_SO_4_–NH_3_ nucleation,^6^ in which neutral and ion-induced nucleation are represented separately. The purely inorganic H_2_SO_4_–NH_3_ pathways retain the same functional form as Dunne *et al.*^6^ whereas the AP-OOM and new H_2_SO_4_-AP-OOM pathways are refitted in the new parameterisation.

### 4.1 Treatment of oxidised organic vapours

Our experimental data involve oxidised organic molecules originating exclusively from α-pinene, which are therefore referred to as AP-OOM. For the parameterisation and its evaluation in the EMAC (ECHAM/MESSy Atmospheric Chemistry) Earth system model, we use the more general variable OOM to represent oxidised organic molecules derived from all monoterpenes. Our measured yield of AP-OOM from α-pinene ozonolysis is 6.0% at +5 °C and 5.7% at −10 °C, while OOM in the EMAC model are derived from monoterpenes with a mean yield of ∼5%.

The good correspondence between the AP-OOM yields measured in CLOUD and the monoterpene-derived oxidised organic yields used in EMAC supports our simple definition of AP-OOM for the present study (see Sections 2.3 and 2.4, and also the SI). Future parameterisations of nucleation rates involving OOM will need further development to account for the influence of other organic precursors, such as isoprene or sesquiterpenes (C_15_H_24_), as well as vapours such as NO_*x*_ and HO_2_, which shift the final products to higher volatility, and reduce the nucleation rates.^7,46,47^

### 4.2 Parameterisation

The new parameterisation developed in this study replaces the Dunne *et al.*^6^ H_2_SO_4_–NH_3_ parameterisation, the Kirkby *et al.*^9^ pure biogenic parameterisation and the Riccobono *et al.*^12^ H_2_SO_4_-BioOxOrg parameterisation. The new parameterisation calculates the total nucleation rate, *J*_total_ (cm^−3^ s^−1^), as the sum of rates from five distinct channels, representing additive, independent contributions to the total nucleation rate:

(1) Neutral nucleation of H_2_SO_4_ (*J*_b,n_);^6^

(2) Neutral nucleation of H_2_SO_4_–NH_3_ (*J*_t,n_);^6^

(3) Neutral nucleation of pure OOM (*J*_0_);

(4) Positive ion-induced nucleation of pure OOM plus pure H_2_SO_4_ (*J*_+_); and

(5) Negative ion-induced nucleation of H_2_SO_4_–NH_3_ (ref. 6) plus H_2_SO_4_–OOM (*J*_−_):

1 *J*_total_ = *J*_b,n_ + *J*_t,n_ + *J*_0_ + *J*_+_ + *J*_−_

(Water is implicit in all these chemical systems.)

The parameters were fitted to 93 nucleation rates measured during the CLOUD15–17 campaigns together with 54 nucleation rates measured by Kirkby *et al.*^9^ The dataset contains measurements at temperatures of −10 °C and +5 °C, ∼60% RH, H_2_SO_4_ concentrations between 10^4^ and 4 × 10^7^ cm^−3^, AP-OOM concentrations between 10^4^ and 6 × 10^8^ cm^−3^, NH_3_ levels below 2 pptv, and three ionisation conditions (neutral, GCR, and pion beam). Temperature-dependent terms in the mixed H_2_SO_4_-OOM pathways are therefore constrained by measurements at only two temperatures, and their application outside this range involves large uncertainties.

Because NH_3_ contaminants were very low (<2 pptv), the parameterisation does not include an explicit NH_3_ dependence for the H_2_SO_4_-OOM pathway, although a strong NH_3_ influence is expected from earlier studies.^14^ For the present study, the influence of NH_3_ remains confined to the H_2_SO_4_–NH_3_ channel inherited from Dunne *et al.*^6^ Future laboratory studies spanning a wider temperature range and including higher NH_3_ concentrations will be required to account for H_2_SO_4_–NH_3_-OOM nucleation over the full range of atmospheric conditions.

**Fig. 5:**
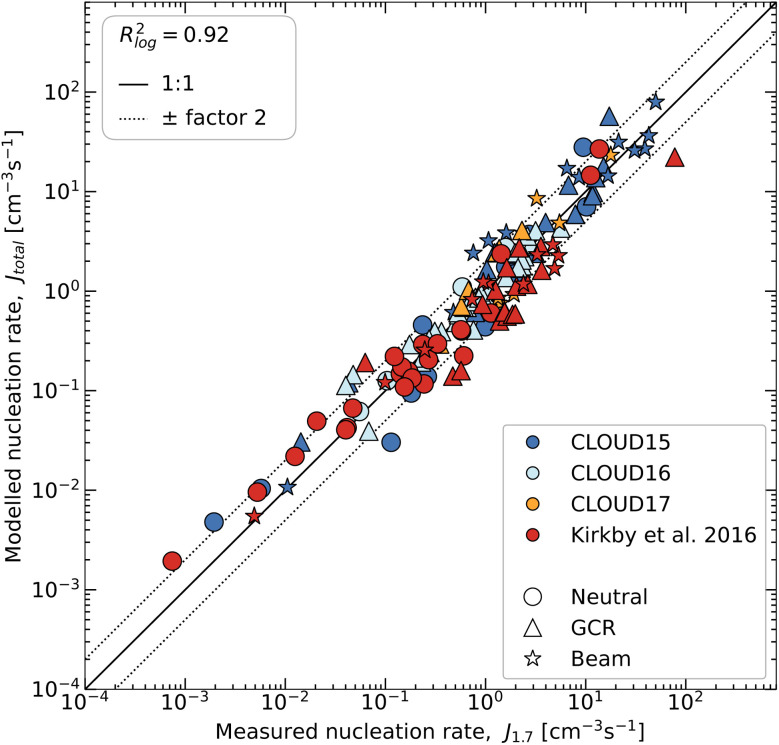
Modelled *versus* measured nucleation rates at −10 °C and +5 °C. The circles correspond to neutral conditions, the triangles to GCR conditions, and the stars to pion beam conditions. The colours correspond to different datasets, as indicated in the legend. The solid line shows a 1 : 1 relationship, and the dotted lines indicate deviations by a factor 2.

Fig. 5 shows the modelled nucleation rates from the H_2_SO_4_-AP-OOM parameterisation *versus* all 147 measured nucleation rates used in the fit. The parameterisation explains more than 90% of the variance in log_10_(*J*_1.7_). It reproduces over 76% of the measured nucleation rates within a factor of two and more than 93% within a factor of three. A full description of the parameterisation equations, fitted coefficients, and model implementation details is provided in the SI.

## 5 Impact on global nucleation rates

**Fig. 6:**
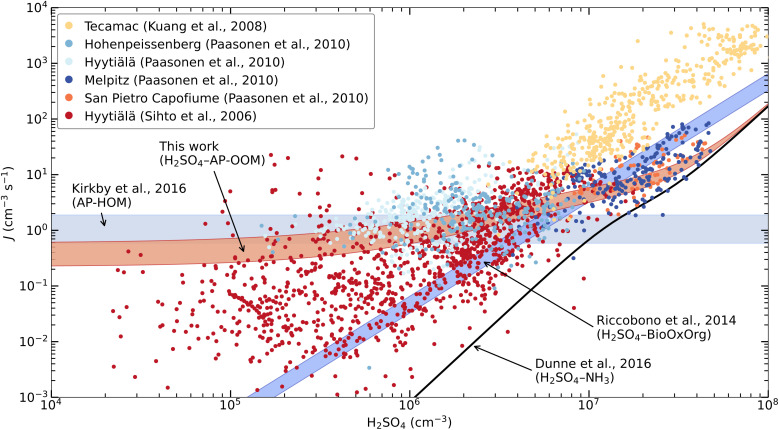
Comparison nucleation rate parameterisations with observations in the boundary layer. The nucleation rates, *J* (cm^−3^ s^−1^), are shown *versus* H_2_SO_4_ concentration. The atmospheric observations^50–52^ are indicated by small coloured circles, and correspond to diverse experimental and environmental conditions. The coloured bands show the parameterisation of the present work (H_2_SO_4_-AP-OOM; red band) together with those from earlier studies: Kirkby *et al.*, 2016 (ref. 9) (AP-HOM; light blue) and Riccobono *et al.*, 2014 (ref. 12) (H_2_SO_4_-BioOxOrg; dark blue), assuming 1–2 × 10^7^ cm^−3^ OOM/HOM/BioOxOrg and −10 °C. The black line shows the parameterisation of Dunne *et al.*, 2016 (ref. 6) for H_2_SO_4_–NH_3_ at 100 pptv NH_3_ and −10 °C.

In Fig. 6 we show a comparison of our nucleation rate parameterisations with observations in the boundary layer. The parameterisations with organic vapours are shown as coloured bands, and assume −10 °C, and 1–2 × 10^7^ cm^−3^ OOM/HOM/BioOxOrg as representative of the cool boundary layer over boreal forests.^48,49^ The parameterisation for H_2_SO_4_–NH_3_ nucleation^6^ is also shown (solid black line), assuming 100 pptv NH_3_ and −10 °C. The atmospheric observations^50–52^ are indicated by small coloured circles and correspond to diverse experimental and environmental conditions. In particular, the Tecamac (Mexico City) measurements^50^ of very rapid nucleation are likely to involve H_2_SO_4_-amine nucleation.^53^ Variable condensation sinks and concentrations of base vapours and NO_*x*_ can account for the large scatter of the nucleation rates measured in less polluted environments.^14^

Fig. 6 shows that the earlier parameterisation of H_2_SO_4_-BioOxOrg^12^ (dark blue band) underestimates nucleation at low H_2_SO_4_ since it accounts neither for pure biogenic nucleation^9^ (light blue band with no dependency on H_2_SO_4_) nor the rise in H_2_SO_4_-AP-OOM synergy at low H_2_SO_4_. On the other, hand, the H_2_SO_4_-BioOxOrg parameterisation overestimates nucleation at high H_2_SO_4_ compared with the present study. The addition of NH_3_ to the H_2_SO_4_-AP-OOM system will tend to shift the threshold for the H_2_SO_4_-AP-OOM(–NH_3_) synergy to lower H_2_SO_4_ concentrations, and raise the nucleation rate above that threshold. The importance of OOM in accounting for nucleation rates in the boundary layer can be readily seen by a comparison with the rates expected for H_2_SO_4_–NH_3_ alone (black line).

**Fig. 7:**
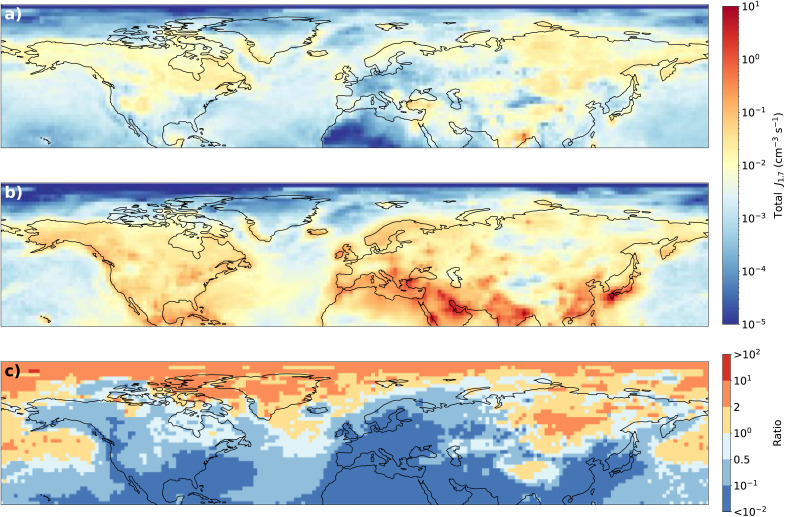
Annually-averaged nucleation rates, *J*_1.7_, at the surface, simulated with the EMAC model. The nucleation rates are calculated using (a) the new parameterisation with H_2_SO_4_-OOM developed in the present study, and (b) the H_2_SO_4_-BioOxOrg parameterisation of Riccobono *et al.*^12^ in combination with the pure biogenic parameterisation of Kirkby *et al.*^9^ Both simulations include H_2_SO_4_–NH_3_ nucleation calculated according to the parameterisation of Dunne *et al.*^6^ Panel (c) shows the ratio of the nucleation rates in panel (a) over those in panel (b).

To estimate its impact on global nucleation rates, we have embedded the new parameterisation in the EMAC (ECHAM/MESSy Atmospheric Chemistry) Earth system model. Fig. 7 compares global nucleation rates (*J*_1.7_) predicted by the new parameterisation with those obtained using the previously implemented scheme combining the H_2_SO_4_-BioOxOrg parameterisation of Riccobono *et al.*,^12^ the pure biogenic Kirkby *et al.*^9^ and the inorganic H_2_SO_4_–NH_3_ parameterisation of Dunne *et al.*^6^ The analysis is restricted to the Northern Hemisphere at ground level, where monoterpene emissions are highest. In the model representation, OOM are produced only from oxidation of monoterpenes; any influence from other biogenic precursors such as isoprene is not included. Fig. 7a shows ground-level nucleation rates calculated using the new parameterisation with H_2_SO_4_-OOM, which is built on the Dunne *et al.*^6^ framework and reverts to this inorganic scheme under purely inorganic conditions. Panel (b) shows nucleation rates obtained using the previously implemented H_2_SO_4_-BioOxOrg scheme^12^ in combination with the pure biogenic parameterisation of Kirkby *et al.*,^9^ and panel (c) displays the ratio of the nucleation rates (new parameterisation over previous).

The new parameterisation introduces several key improvements compared with the previous parameterisations. First, consistent with the treatment of organic vapours in the EMAC model, it represents oxidised organic vapours based on monoterpene oxidation, rather than relying on proxy species like BioOxOrg.^12^

Second, it includes a temperature dependence of the mixed organic–inorganic nucleation pathway, which was not available previously. Nevertheless, we caution that the temperature dependence is constrained by measurements at only two temperatures (−10 °C and +5 °C) so its application outside this range is uncertain.

The new parameterisation predicts higher nucleation rates over boreal forests, where monoterpene-derived OOM and H_2_SO_4_ coexist in the boundary layer under cool conditions (Fig. 7c). When the effects of NH_3_ are also included, the nucleation rates are expected to be further enhanced.^14^ In these boreal forest regions, the H_2_SO_4_-BioOxOrg parameterisation^12^ underestimated NPF rates. This can be seen in Fig. 6 at 1–5 × 10^6^ cm^−3^ H_2_SO_4_ and is even more pronounced at lower AP-OOM/BioOxOrg.

On the other hand, the new parameterisation predicts lower nucleation rates at lower latitudes of the Northern Hemisphere, where higher temperatures suppress nucleation despite the presence of precursor vapours. The earlier scheme, without any temperature dependence, strongly overestimates nucleation rates at lower latitudes. The strength of the decrease seen in (Fig. 7c) varies regionally since it depends on not only the local temperature but also the H_2_SO_4_, OOM and NH_3_ concentrations. The largest reductions occur where OOM and H_2_SO_4_ coexist at appreciable concentrations under warm conditions, for which the previous temperature-independent H_2_SO_4_-BioOxOrg parameterisation predicts rapid nucleation. Once more, this can be seen in Fig. 6 at H_2_SO_4_ concentrations above about 10^7^ cm^−3^. The discrepancies are smaller where OOM or H_2_SO_4_ are limiting; for these conditions the nucleation rate largely depends on the pure inorganic or pure biogenic systems, respectively, which are reasonably well parameterised.

Recent aircraft observations have shown that biogenic monoterpenes can persist at mixing ratios of a few tens of pptv in the upper troposphere, for example over the Amazon basin.^54^ In that study, new particle formation was associated with highly oxidised organic products from isoprene. These observations indicate that biogenic organic precursors and their oxidation products may be present well above the surface and can contribute to particle formation at high altitudes. In regions where monoterpene emissions dominate, such as boreal forests, oxidation products from monoterpenes may therefore also contribute to new particle formation through mixed organic–H_2_SO_4_ pathways, especially at cold, high altitudes, even when the concentrations of the individual vapours alone would be insufficient to produce substantial nucleation rates.

## 6 Conclusions

We have investigated new particle formation from α-pinene oxidation products (AP-OOM) and H_2_SO_4_ in the CERN CLOUD chamber. Through controlled experiments at atmospheric AP-OOM and H_2_SO_4_ concentrations, we have quantified the synergistic interaction between these two vapours in a near NH_3_-free environment (NH_3_ contamination below 2 pptv). We conducted measurements at −10 °C and +5 °C under variable ionisation conditions representative of the boundary layer and the free troposphere.

Our results demonstrate that small additions of H_2_SO_4_ strongly enhance AP-OOM-driven nucleation and, similarly, that small AP-OOM concentrations strongly enhance H_2_SO_4_-driven nucleation, where “small” implies levels that would produce negligible nucleation alone. This behaviour cannot be explained by inorganic H_2_SO_4_–NH_3_ nucleation, indicating a synergistic interaction between AP-OOM and H_2_SO_4_ rather than simply the addition of two independent nucleation mechanisms. The enhancement is more pronounced at lower temperatures, reflecting increased cluster stability under colder conditions. Across the full tropospheric ionisation conditions, nucleation rates are higher with increasing ionisation rates, indicating the importance of ion-induced nucleation for H_2_SO_4_-AP-OOM, as well as for pure AP-OOM and H_2_SO_4_(–NH_3_).

Based on our measurements, we have developed a new parameterisation describing neutral and ion-induced nucleation in the H_2_SO_4_-AP-OOM system, built on the H_2_SO_4_(–NH_3_) parameterisation of Dunne *et al.*^6^ at low AP-OOM. The parameterisation reproduces the measured nucleation rates within a factor of around two and captures their dependence on temperature, organic vapour concentrations, and ionisation.

When implemented in the EMAC global model, the new parameterisation predicts enhanced nucleation rates over high-monoterpene regions of the Northern Hemisphere compared with the earlier H_2_SO_4_-BioOxOrg parameterisation of Riccobono *et al.*^12^ Recent observations indicating the presence of monoterpenes at high altitudes suggest that the mixed H_2_SO_4_-AP-OOM pathway identified here may also be relevant at higher altitudes.

The present experiments involve H_2_SO_4_-AP-OOM nucleation under ultra-low NH_3_ conditions and at −10 °C and +5 °C. Further experiments spanning a wider temperature range, relative humidities and atmospheric NH_3_ concentrations will be required to extend this work to encompass the four-component H_2_SO_4_-AP-OOM-NH_3_–H_2_O system. Moreover, future parameterisations of nucleation rates involving OOM will need to account for more complex chemical environments that affect the volatility spectrum, combining monoterpenes with isoprene, sesquiterpenes, NO_*x*_ and HO_2_.

## Author contributions

E. S., J. A., M. S., L. D., T. P., D. M. R., U. B., J. C., N. M. D., I. E. H., H. J., M. Ku., K. L., S. S., D. R. W. and J. K. planned the experiments; E. S., J. A., M. S., R. B., J. Du., P. K., A. Pi., P. R., S. Ru., A. A., H. J. and A. T. developed software; C. X., A. Po., M. Ko. and T. C. conducted the model implementation; E. S., J. A., W. Y., M. S., F. M., C. X., Z. Z., L. C.-P., M. L., L. L., M. M., T. R., D. M. R., Y. T., T. C. and J. K. analysed the data; E. S., J. A., W. Y., M. S., F. M., C. X., Z. Z., N. B., D. A., S. A., R. B., H. B., M. Be., P. B., M. Bu., M. C., L. C.-P., A. C., R. C.-S., L. D., J. De., J. Du., M. G., L. G. S., H. G. H., E. H., A. J., B. J., M. K. S., H. K., P. K., R. K., T. K., F. K., M. L., C. J. L., L. L., R. M., B. M., A. M., M. M., A. Pi., P. R., T. R., S. R., B. R., S. Ru., D. M. R., W. S., J. S., A. S., R. C. T., Y. T., J. T., N. S. U., J. W., B. Y., M. Z.-W., J. Z., T. C., H. H., X.-C. H., H. J., R. V., P. M. W. and J. K. conducted the experiments; E. S. and J. K. wrote the manuscript; E. S., J. A., W. Y., M. S., C. X., A. P., Z. Z., N. B., J. De., H. G., H. G. H., H. K., M. K., F. K., W. S., U. B., J. C., N. M. D., I. E. H., R. C. F., X.-C. H., K. L., J. L., S. S., P. M. W. and J. K. commented on and edited the manuscript; E. S., J. A., F. M., X. C. and T. C. visualised the data; H. G., A. K., R. M., T. C., J. C., A. H., H. J., M. Ku., K. L., J. L., S. S., R. V., P. M. W. and J. K. provided supervision; E. S., T. P., J. C., N. M. D., I. E. H., X.-C. H., H. J., M. Ku., R. V., A. O. and J. K. managed the project; J. Du., H. G., T. P., U. B., T. C., J. C., N. M. D., I. E. H., R. C. F., A. H., H. J., M. Ku., K. L., J. L., O. M., S. S., R. V. A. O. and J. K. acquired funding.

## Conflicts of interest

There are no conflicts to declare.

## Supplementary Material

EA-006-D6EA00046K-s001

EA-006-D6EA00046K-s002

EA-006-D6EA00046K-s003

EA-006-D6EA00046K-s004

EA-006-D6EA00046K-s005

EA-006-D6EA00046K-s006

EA-006-D6EA00046K-s007

## Data Availability

The data is available on Zenodo under this DOI: 10.5281/zenodo.19350026. Supplementary information (SI) is available. See DOI: https://doi.org/10.1039/d6ea00046k.
