# Supplementary material for: Nucleation of α-pinene oxidation products with sulfuric acid
Source: Environ Sci Atmos. 2026 Jun 9. Online ahead of print. doi: 10.1039/d6ea00046k (PMC13285975; doi:10.1039/d6ea00046k)
Supplement: EA-OLF-D6EA00046K-s001 [file EA-OLF-D6EA00046K-s001.pdf]

Supplementary Information (SI) for Environmental Science: Atmospheres.
This journal is © The Royal Society of Chemistry 2026

Supporting Information for Nucleation of α-pinene oxidation products with sulfuric acid

Eva Sommer*[a,b], João Almeida[a,c], Wenjuan Yu[d], Mario Simon[e], Felix Möller[f], Christos Xenofontos[g], Andrea Pozzer[g,h], Zhensen Zheng[i,j], Nirvan Bhattacharyya[e], Dina Alfaouri[d], Samira Atabakhsh[k], Rima Baalbaki[g], Hannah Beckmann[f], Moritz Berntheusel[e], Pia Bogert[l], Mattia Busato[a], Manjula Canagaratna[m], Luía Caudillo-Plath[e], Anouck Chassaing[n,o], Romulo Cruz-Simbron[p,q], Lubna Dada[r], Jenna DeVivo[s,t], Jonathan Duplissy[d,u], Hamish Gordon[v,t], Manuel Granzin[e], Lena Große Schute[e], Herbert G. Hartl[d,w], Ella Hirvensalo[d], Aenne Jacobshagen[b], Bernhard Judmaier[i], Milin Kaniyodical Sebastian[l], Hannah Klebach[e], Paap Koemets[f,x], Matthias Kohl[h], Ruth Konrat[b], Timm Krüger[e], Felix Kunkler[h], Andreas Kürten[e], Markus Leiminger[j], Clara J. Lietzke[p,q], Lu Liu[r], Roy Mauldin[s,y], Bernhard Mentler[i], Aleksandra Morawiec[b], Markus Müller[j], Tuukka Petäjä[d], Alessia Pignatelli[r], Pedro Rato[a,e], Tobias Reinecke[j], Sarah Richter[e], Birte Rörup[d], Samuel Ruhl[h], Douglas M. Russell[e], Wiebke Scholz[i], Jiali Shen[d,u], Alexandria Stinchfield[v,t], Roseline C. Thakur[d], Yandong Tong[p,q], Jens Top[r,d], Nsikanabasi Silas Umo[z], Jakob Weissbacher[i], Boxing Yang[r], Marcel Zauner-Wieczorek[e], Jiangy Zhang[d], Antonio Amorim[c,α], Urs Baltensberger[r], Theodoros Christoudias[g], Joachim Curtius[e], Neil M. Donahue[v,s,t,β], Imad El Haddad[r,δ], Richard C. Flagan[γ], Armin Hansel[i], Hartwig Harder[h], Xu-Cheng He[d], Heikki Junninen[ϵ], Markku Kulmala[d], Katrianne Lehtipalo[d,ζ], Jos Lelieveld[h,g], Ottmar Möhler[l], Siegfried Schobesberger[η], António Tomé[θ], Rainer Volkamer[p,q], Douglas R. Worsnop[d,m], Antti Onnela[a], Paul M. Winkler[b], and Jasper Kirkby*[a,e]

[a] CERN, European Organization for Nuclear Research, 1211 Geneva, Switzerland
[b] Faculty of Physics, University of Vienna, Strudlhofgasse 4, 1090 Vienna, Austria
[c] LIP - Laboratory for Instrumentation and Experimental Particle Physics, Av. Prof. Gama Pinto, n.2, 1649-003 Lisboa, Portugal
[d] Institute for Atmospheric and Earth System Research/Physics, Faculty of Science, University of Helsinki, 00014 Helsinki, Finland
[e] Goethe University Frankfurt, Institute for Atmospheric and Enviromental Sciences, Altenhoeferallee 1, 60438 Frankfurt am Main, Germany
[f] Institute of Physics, Faculty of Science and Technology, University of Tartu, 50411, Tartu, Estonia
[g] Climate and Atmosphere Research Center, The Cyprus Institute, 1645 Nicosia, Cyprus
[h] Atmospheric Chemistry Department - Max Planck Institute for Chemistry, Hahn-Meitner-Weg 1, 55128 Mainz, Germany
[i] Institute for ion and applied physics, University of Innsbruck, Technikerstraße 25, 6020 Innsbruck, Austria
[j] IONICON Analytik GmbH, 6020 Innsbruck, Austria
[k] Leibniz-Institute for Tropospheric Research, Permoser Straße 15, 04318 Leipzig, Germany
[l] Institute of Meteorology and Climate Research, Karlsruhe Institute of Technology, Karlsruhe, Germany
[m] Aerodyne Research, Billerica, MA 01821, USA
[n] Department of Environmental Science, Stockholm University, Stockholm, 10961 Sweden
[o] Bolin Centre for Climate Research, Stockholm University, Stockholm, 10961 Sweden
[p] Department of Chemistry, University of colourado Boulder, Cristol Chemistry, 215 UCB, Boulder, CO, USA
[q] CIRES, Cooperative Institute for Research in Environmental Sciences, University of colourado Boulder, Boulder, CO 80309-0215, USA
[r] PSI Center for Energy and Environmental Sciences, Paul Scherrer Institute, 5232 Villigen PSI, Switzerland
[s] Department of Chemistry, Carnegie Mellon University, 5000 Forbes Ave Pittsburgh, PA 15213 USA
[t] Center for Atmospheric Particulate Studies, Carnegie Mellon University, 5000 Forbes Ave Pittsburgh, PA 15213 USA
[u] Helsinki Institute of Physics, University of Helsinki, Helsinki, Finland
[v] Department of Chemical Engineering and Center for Atmospheric Particle Studies, Carnegie Mellon University, Pittsburgh, PA 15213, USA
[w] Airmodus Oy., Erik Palmenin aukio 1, 00560 Helsinki, Finland
[x] Airel Ltd, Observatooriumi 5, 61602 Tõravere, Estonia
[y] Department of Atmospheric and Oceanic Sciences, University of colourado, Boulder, Boulder, CO 80309, USA
[z] Department of Chemistry and Biochemistry, University of North Carolina Wilmington, NC 28403, USA
[α] Faculdade de Ciencias da Universidade de Lisboa, Edificio C8, Campo Grande, 1749-016 Lisboa, Portugal
[β] Department of Engineering and Public Policy, Carnegie Mellon University, 5000 Forbes Ave, Pittsburgh, PA 15213

USA
[γ] California Institute of Technology, Pasadena, CA 91125, USA
[δ] College of Environmental Sciences and Engineering, Peking University, Beijing 100871, China
[ϵ] Laboratory of Atmospheric and Environmental Sciences, University of Tartu, W. Ostwaldi tn 1, 50411, Tartu, Estonia
[ζ] Finnish Meteorological Institute, Helsinki, Finland
[η] Department of Technical Physics, University of Eastern Finland, PO Box 1627, 70211 Kuopio, Finland
[θ] IDL-Universidade da Beira Interior, Rua Marquês D'Ávila e Bolama 6201-001 Covilhã, Portugal

1 Precursor gas and condensable vapour measurements

1.1 α-Pinene measurements

During the CLOUD15-17 measurement campaigns we measured α-pinene concentrations using a H_3O-PTR-MS (STOF)[1;2] with a limit of detection of around 150 pptv. During CLOUD16, α-pinene was monitored with a FUSION-PTR-MS[3] in addition to the STOF. The FUSION-PTR has a significantly lower detection limit (∼0.3 pptv for 1 min averaging) than the STOF. At concentrations above 200 pptv, the STOF and the FUSION PTR-MS showed good agreement, and both instruments correlated linearly with the α-pinene injection rate (AP_{inj} [ml min^{-1}]) determined from the mass flow controller (MFC) settings. At lower concentrations, the STOF signal deviated due to its higher limit of detection, whereas the FUSION instrument retained linearity with the MFC-derived injection rate. As shown in Fig. S1, we fitted a linear function to the FUSION measurements (supported by simultaneous STOF data at $>$ 200 pptv) and used the α-pinene injection rate, AP_{inj} to derive α-pinene concentrations $[AP]_{Calc}$ for experiments where only STOF measurements were available:

$$[AP]_{Calc}(pptv) = 1.80 \cdot 10^3 \cdot AP_{inj}(ml\ min^{-1}) \tag{S1}$$

1.2 H_2SO_4 and AP-OOM measurements

We used a nitrate chemical ionisation atmospheric pressure interface time-of-flight mass spectrometer (NO_3-CI-APi-TOF, hereafter nitrate-CIMS) to measure sulfuric acid (H_2SO_4)[4] and highly oxidised organic molecules from α-pinene (AP-OOM)[5]. A H_2SO_4 calibration[6;7] was performed in each campaign using the calibration method described in Kürten *et al.*[7]. The calibration factors were $6.15 \cdot 10^9$ cm^{-3} for CLOUD15 and $1.06 \cdot 10^{10}$ cm^{-3} for CLOUD17. For CLOUD16 two calibration factors were determined at the beginning and the end of the experiments, with values of $1.85 \cdot 10^{10}$ cm^{-3} and $1.25 \cdot 10^{10}$ cm^{-3} respectively. A comparison with independent H_2SO_4 measurements by the BrMION instrument[8] suggests a calibration factor at 74 % of the latter value ($1.25 \cdot 10^{10}$ cm^{-3}). Therefore, we applied a final H_2SO_4 calibration factor of $1.25 \cdot 10^{10}$ cm^{-3} with an uncertainty range of 74-148 % which lies within the overall uncertainty of the measurement of a factor of two.

The data processing workflow included normalization of the raw ion signals to the nitrate reagent ions ($(HNO_3)_nNO_3^-$, n = 0–2, and $(H_2O)NO_3^-$), correction for the mass-dependent transmission efficiency[9], and a sampling-line loss correction following Simon *et al.*[10]. Application of the H_2SO_4 calibration factor yielded absolute concentrations. Signals from analytes detected as clusters with multiple reagent ions, were summed to obtain the total concentration.

In the absence of compound-specific calibrations, many studies quantify oxidized organics measured by nitrate-CIMS using the H_2SO_4 calibration factor, assuming near-collision-limited charging (kinetic limit) and similar transmission efficiencies to sulfuric acid[11;12]. We therefore apply the same calibration factor to AP-OOM as for H_2SO_4. The overall systematic uncertainty of the quantification of H_2SO_4 is about a factor of two, while the relative point-to-point uncertainty is in the range of ± 30 %. Because there is no direct calibration for these species, the quantification

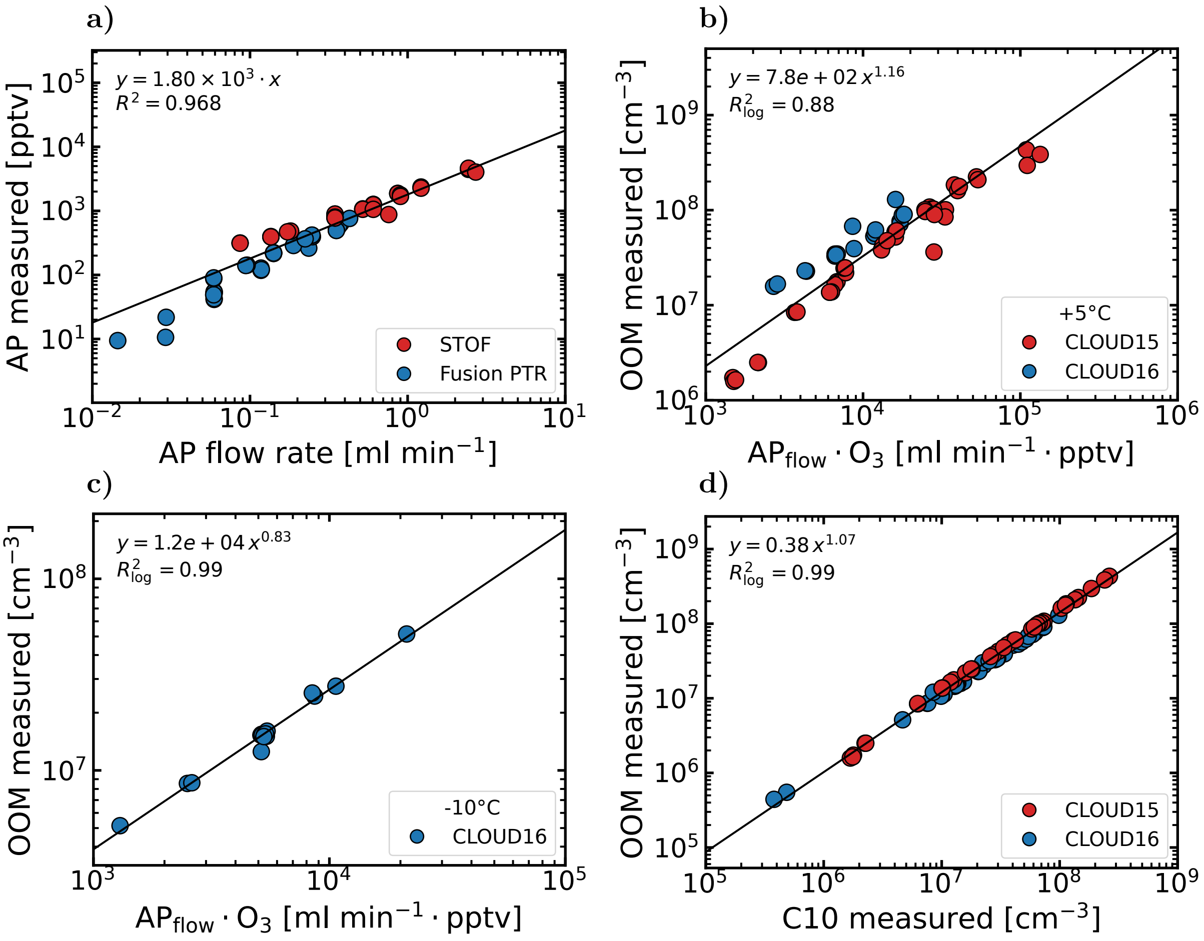

Figure S1: Estimate of steady-state concentrations of organic precursor gases in the CLOUD chamber. Panel a) shows α-pinene (AP) concentrations measured by the STOF and Fusion PTR vs the gas flow from the α-pinene evaporator system. Values below 200 pptv measured by the STOF are not shown and excluded from the linear fit. Panels b) and c) show the AP-OOM concentrations measured by the nitrate-CIMS plotted against the AP flow rate multiplied with the measured steady-state O_3 concentration at +5 °C and -10 °C respectively. Panel d) shows the corellation of measured AP-OOM concentrations vs the measured C10 species.

of AP-OOM has a higher uncertainty. Especially less functionalised organic compounds may have lower charging efficiencies by the nitrate reagent ions. Therefore, any given AP-OOM concentration should be interpreted as a lower limit estimate.

1.3 AP-OOM definition in experimental and modelling sections

In this work, AP-OOM are operationally defined as organic species detected by the nitrate-CIMS, following the definition of Wang *et al.*[12]. No additional constraints (minimum carbon number or O:C ratio) are applied. We filter the signal according to elemental composition to isolate organic oxidation products. Since no NOx was present in the chamber, the detected OOM consist exclusively of $C_xH_yO_z$ compounds. α-Pinene was the only injected organic precursor, so we refer to these species as AP-OOM.

For AP-OOM we determined a total loss rate in the chamber of $1.88 \cdot 10^{-3}$ s^{-1} at +5 °C and $1.80 \cdot 10^{-3}$ s^{-1} at -10 °C. The AP-OOM yield from α-pinene was then derived following the approach of Simon *et al.*[13], using the measured steady-state concentrations and chamber loss rates, resulting in an average yield of approximately 6 %. To assess consistency with the organic vapour representation used in the EMAC model, we compared the CLOUD AP-OOM definition to the ELVOC definition

introduced by Jokinen *et al.*[14], which underlies the organic vapour representation in EMAC. Jokinen *et al.*[14] define ELVOC from α-pinene as nitrate-CIMS-detected species within the mass range 280–620 Th, with reported yields of 3.4 % for O_3-initiated oxidation and 0.44 % for OH-initiated oxidation. Applying the Jokinen *et al.*[14] mass range criterion (280–620 Th) to the CLOUD AP-OOM dataset retains approximately 91 % of the detected AP-OOM signal at both temperatures. The resulting effective CLOUD AP-OOM yield is therefore ∼5.5 %. In the CLOUD experiments, AP-OOM production includes contributions from both O_3- and OH-initiated oxidation of α-pinene, with additional OH production under UV illumination. Accounting for these contributions results in an effective Jokinen-based ELVOC yield of approximately 4.0 %, which is in close agreement with the CLOUD-derived value. Given the close agreement in effective yields and the small impact of the ELVOC mass-range restriction (∼10 %), we treat ELVOC as representative of OOM under the conditions considered here and use the more general term OOM for consistency with the EMAC implementation.

We conclude that the AP-OOM concentrations derived from CLOUD measurements are quantitatively consistent with the ELVOC representation used in EMAC. While applying an explicit mass-range filter would slightly reduce AP-OOM concentrations (by ∼10 %), this correction is small relative to other uncertainties and is not applied in the present parameterisation.

1.4 AP-OOM corrections

AP-OOM concentrations were corrected for instrument sensitivity variations between measurement campaigns. To obtain campaign-consistent AP-OOM concentrations, we established empirical relationships between measured nitrate-CIMS AP-OOM concentrations and a proxy for the α-pinene ozonolysis rate, defined as [α-pinene] × [O_3].

For each temperature (-10 °C and +5 °C), we plotted measured AP-OOM concentrations from CLOUD15–17 against this proxy. As shown in Fig S1, panels b) and c), we fitted power-law relationships of the form $a \cdot ([AP] \cdot [\mathrm{O_3}])^b$ to the data, with the intercept constrained to zero. The resulting expressions (equations S2 and S3) were used to calculate AP-OOM concentrations from [α-pinene] × [O_3] for all experiments in these campaigns.

$$[AP\text{-}OOM]_{T=+5^{\circ}C}(cm^{-3}) = 1.10 \cdot 10^3 \cdot ([AP]_{Calc} \cdot [\mathrm{O_3}])^{1.12}, (R^2 = 0.93) \tag{S2}$$

$$[AP\text{-}OOM]_{T=-10^{\circ}C}(cm^{-3}) = 1.20 \cdot 10^4 \cdot ([AP]_{Calc} \cdot [\mathrm{O_3}])^{0.83}, (R^2 = 0.99) \tag{S3}$$

For earlier experiments reported in Kirkby *et al.*[15], direct AP-OOM measurements were not available. To extend the approach to these data, we used the concentration of C10 organic species measured by the nitrate-CIMS, which were available in both the earlier and more recent CLOUD datasets. In the CLOUD15–17 data, C10 concentrations correlate strongly with measured AP-OOM concentrations (Figure S1, panel d)), indicating that they provide a robust proxy for AP-OOM under the experimental conditions considered here. We therefore derived an analogous power-law relationship between C10 and AP-OOM (equation S4) and used this expression to estimate AP-OOM concentrations for the Kirkby *et al.* dataset.

$$[AP\text{-}OOM]_{T=+5^{\circ}C}(cm^{-3}) = 0.38 \cdot ([C10]_{meas})^{1.07}, (R^2 = 0.99) \tag{S4}$$

2 Particle growth rates

We measured the growth rates of aerosol particles between 3 and 8 nm with a Neutral cluster and Air Ion Spectrometer (NAIS)[16] throughout the CLOUD15–17 campaigns and also with a DMA-train[17] during CLOUD15. A comparison of the rates measured with the two instruments indicates good agreement. Fig. S2 shows the growth rates versus AP-OOM + H_2SO_4, with the colour indicating H_2SO_4 concentration. Within systematic uncertainties, the growth rates agree with those previously reported by Stolzenburg *et al.*[18] at 5 °C.

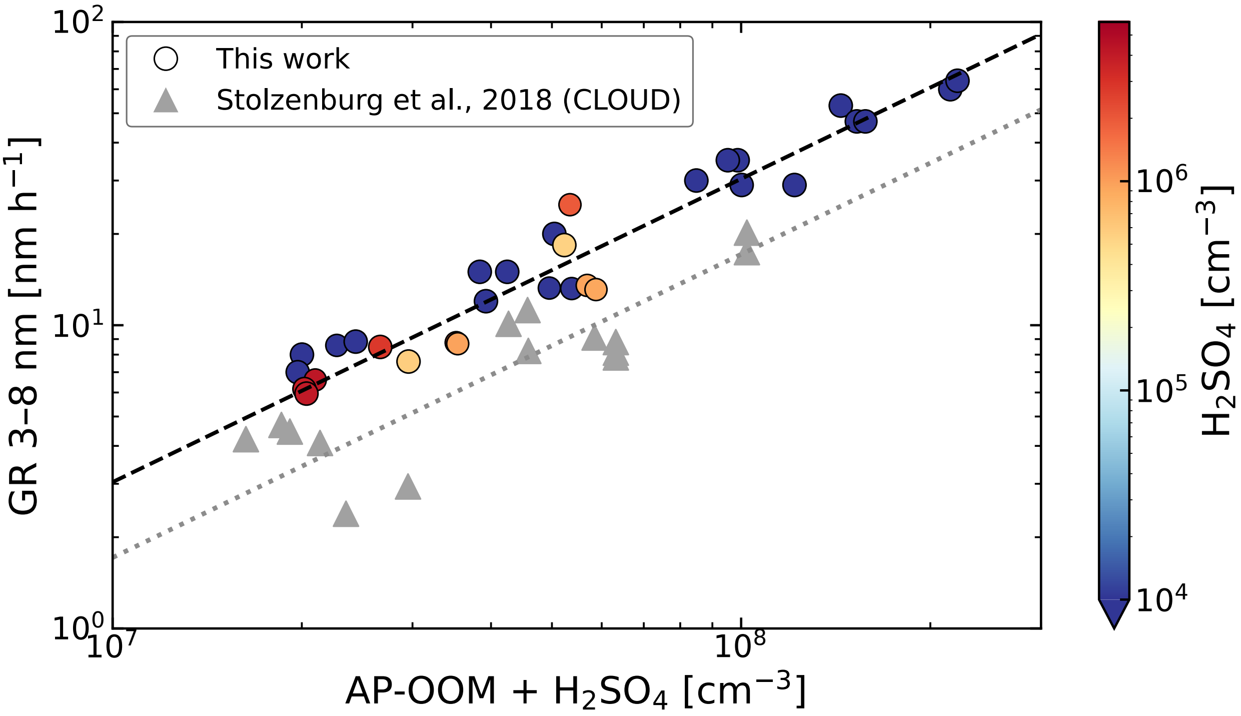

Figure S2: Particle growth rates versus AP-OOM + H_2SO_4. The growth rates (GRs) are measured from 3 to 8 nm particle size and are coloured by H_2SO_4 concentration. The circles show growth rates measured in this study, while the grey triangles show growth rates measured at +5 $°C$ from Stolzenburg *et al.*[18]. The dashed and dotted lines represent linear fits through the origin for each dataset. The data with no H_2SO_4 added to the chamber are set to 10^4 cm^{-3}, corresponding to the instrument detection limit. Both the present study and Stolzenburg *et al.*[18] use the same instrument (nitrate-CIMS) for the measurement of AP-OOM. The experimental conditions are -10 °C and +5 °C, 40-80 ppbv O_3, <2 pptv NH_3, 80–4000 pptv α-pinene and 60 % RH.

3 Parameterisation of nucleation rates from H_2SO_4 and AP-OOM

3.1 Organic vapour representation

In the experimental sections of this study, we refer to the oxidised organic compounds as AP-OOM, reflecting that they originate exclusively from α-pinene oxidation in the CLOUD chamber. In the parameterisation framework, we use the more general symbol OOM to denote the concentration of oxidised organic vapours. For the dataset underlying the fit, OOM corresponds directly to the experimentally derived AP-OOM concentrations defined above.

The use of the more general OOM notation reflects the intended model application, in which oxidised organics are derived from bulk monoterpene emissions. As discussed above, the AP-OOM yields measured in CLOUD are quantitatively consistent with the monoterpene-derived ELVOC

yields implemented in EMAC. In model applications, OOM therefore represents monoterpene-derived highly oxidised organic vapours. Application of the parameterisation to systems with substantial contributions from other organic precursors, such as isoprene, may introduce additional uncertainties and is not constrained by the present dataset.

3.2 Inputs and structure

The parameterisation represents the total particle formation rate J_{total} as the sum of five physically distinct pathways:

$$J_{total} = J_{b,n} + J_{t,n} + J_0 + J_+ + J_- \tag{S5}$$

where $J_{b,n}$ and $J_{t,n}$ denote the binary and ternary neutral inorganic pathways inherited from Dunne *et al.*[19], J_0 represents neutral OOM-related pathways, and J_+ and J_- represent positive and negative ion-induced pathways. An overview of the mathematical representation of these pathways is shown in Table S1 and all fitted exponents are listed in Table S2.
The model requires the following environmental inputs: temperature T, trace gas concentrations ($[H_2SO_4]$, $[NH_3]$, and [OOM]), and the ion-pair production rate Q. The scheme includes (i) a vector of fixed parameters ('aD') inherited from the CLOUD-based parameterisation of Dunne *et al.*[19] and (ii) a set of twelve fitted parameters a_i (Table S2) optimised in the present study. The fixed parameters define pathways involving only H_2SO_4 and NH_3, while the fitted parameters control the magnitude and dependencies of OOM-related pathways as well as the growth-time scaling used in the calculation of survival probabilities.

Because NH_3 concentrations in the fitting dataset were consistently low (< 2 pptv), NH_3-dependent behaviour in mixed AP-OOM–H_2SO_4 nucleation pathways could not be constrained. Consequently, no additional NH_3 dependence was introduced in the OOM-related pathways, and all NH_3 effects remain defined by the fixed inorganic formulation of Dunne *et al.*[19].

3.3 Summary of pathway equations

To support model implementation, we summarise here the analytical expressions used for all OOM-related nucleation pathways that were developed in this work. The purely inorganic neutral pathways ($J_{b,n}$ and $J_{t,n}$) and the detailed formulation of the inorganic negative-ion terms ($ecVal$ and $kbcVal$) are inherited unchanged from the fixed CLOUD-based parameter set 'aD' of Dunne *et al.*[19] and are not reproduced here. A description of all Dunne *et al.*[19] parameters at full numerical precision is provided in Gordon *et al.*[20].

The parameters fitted in this work are listed in Table S2 and enter the pathway equations as follows.

- **Neutral pure biogenic pathway ($J_{0,OOM}$).** The fitted prefactor $A_{0,OOM}$ and the associated OOM exponent determine the magnitude and OOM-dependence of the neutral OOM-only pathway.
- **Neutral mixed H_2SO_4–OOM pathway ($J_{0,mix}$).** This pathway uses fitted power-law dependencies on OOM and $[H_2SO_4]$ with exponents $\gamma_{OOM,0,mix}$ and $\gamma_{SA,0,mix}$, together with a temperature-dependent prefactor of the form $exp(A_{0,mix} - B_{0,mix} \cdot T)$.
- **Positive ion OOM-only initiation term ($J_{+,OOM}$).** The magnitude and OOM-dependence of the positive ion OOM-only initiation pathway are controlled by the fitted exponent $\gamma_{OOM,+,OOM}$ and the prefactor $exp(A_{+,OOM})$.

- **Mixed ion-induced H_2SO_4–OOM pathway ($J_{Ion,mix}$).** This pathway includes a temperature-dependent prefactor defined by $A_{Ion,mix}$ and $B_{Ion,mix}$, a power-law dependence on $[H_2SO_4]$ with exponent $\gamma_{Ion,mix}$, and a saturating dependence on [OOM] governed by the fixed constant $K_{OOM}^{Ion,mix}$ (equation S16). The same $J_{Ion,mix}$ formulation is used in both the positive and negative ion-induced nucleation pathways, with polarity-specific effects entering through the ion balance and survival probabilities.
- **Growth-time scaling.** The fitted parameter C_t enters the definition of the cluster growth time (equation S7) and thereby influences the survival probabilities $P_{surv}^{+,-}$ used in the ion-induced pathways.

$\boldsymbol{J_{\mathrm{total}} = J_{b,n} + J_{t,n} + J_0 + J_+ + J_-}$	
$\boldsymbol{J_0} = J_{0,\mathrm{OOM}} + J_{0,\mathrm{mix}}$	$\boldsymbol{J_{0,\mathrm{OOM}}} = \exp(A_{0,\mathrm{OOM}}) \cdot [\mathrm{OOM}]^{\gamma_{0,\mathrm{OOM}}}$<br>$\boldsymbol{J_{0,\mathrm{mix}}} = \exp(A_{0,\mathrm{mix}} - B_{0,\mathrm{mix}} \cdot T) \cdot [\mathrm{OOM}]^{\gamma_{\mathrm{OOM},0,\mathrm{mix}}} \cdot [\mathrm{H_2SO_4}]^{\gamma_{\mathrm{SA},0,\mathrm{mix}}}$
$\boldsymbol{J_+} = n_{\mathrm{ss}}^{+} \cdot (J_{+,\mathrm{OOM}} + J_{\mathrm{Ion,mix}}) \cdot P_{\mathrm{surv}}^{+}$	$\boldsymbol{J_{+,\mathrm{OOM}}} = \exp(A_{+,\mathrm{OOM}}) \cdot [\mathrm{OOM}]^{\gamma_{+,\mathrm{OOM}}}$<br>$\boldsymbol{J_{\mathrm{Ion,mix}}} = \exp(A_{\mathrm{Ion,mix}} - B_{\mathrm{Ion,mix}} \cdot T) \cdot \frac{[\mathrm{OOM}]}{K_{\mathrm{OOM}}^{\mathrm{Ion,mix}} + [\mathrm{OOM}]} \cdot [\mathrm{H_2SO_4}]^{\gamma_{\mathrm{Ion,mix}}}$
$\boldsymbol{J_-} = J_{-,\mathrm{SA}} + J_{\mathrm{Ion,mix}} \cdot n_{\mathrm{ss}}^{-} \cdot P_{\mathrm{surv}}^{-}$	$\boldsymbol{J_{-,\mathrm{SA}}} = J_{b,i} + J_{t,i}$<br>*(same $J_{\mathrm{Ion,mix}}$ as in J_+)*

Table S1: Decomposition of the total formation rate J_{total} into pathway-level expressions (left column) and component terms (right column).

**Symbol**	**Pathway**	**Description**	**Source (a_i)**	**Approx. value**
$\gamma_{0,OOM}$	$J_{0,OOM}$	OOM exponent	a_1	1.78
$\gamma_{OOM,0,mix}$	$J_{0,mix}$	OOM exponent	a_2	2.06
$\gamma_{SA,0,mix}$	$J_{0,mix}$	H_2SO_4 exponent	a_3	0.74
$\gamma_{+,OOM}$	$J_{+,OOM}$	OOM exponent	a_4	1.05
C_t		Growth time numerator	a_5	$2.71 \cdot 10^9\ s\ cm^{-3}$
$\gamma_{Ion,mix}$	$J_{Ion,mix}$	H_2SO_4 exponent	a_6	1.15
$A_{0,mix}$	$J_{0,mix}$	Log-prefactor	a_7	-26.63
$A_{0,OOM}$	$J_{0,OOM}$	Log-prefactor	a_8	-32.33
$A_{Ion,mix}$	$J_{Ion,mix}$	Log-prefactor	a_9	21.53
$A_{+,OOM}$	$J_{+,OOM}$	Log-prefactor	a_{10}	-24.29
$B_{0,mix}$	$J_{0,mix}$	T-coefficient	a_{11}	$0.071\ K^{-1}$
$B_{Ion,mix}$	$J_{Ion,mix}$	T-coefficient	a_{12}	$0.173\ K^{-1}$

Table S2: Fitted parameters used in the OOM-related nucleation pathways and growth-time parameterisation. All other quantities are fixed constants or inherited from the 'aD' parameter set of Dunne *et al.*[19].

3.4 Derivation and definition of shared intermediate quantities

The following intermediate quantities are used across multiple pathways and represent basic physical processes: the steady-state ion concentrations $n_{ss}^{+,-}$, the cluster growth time t_{growth}, the effective cluster loss frequency $1/\tau_{loss}^{+,-}$, and the survival probability $P_{surv}^{+,-}$. The ion steady-state formulation follows the same conceptual form as in Dunne *et al.*[19], while the pathway-dependent loss frequencies $b^{+,-}$ defined below incorporate the OOM-related initiation terms used in the present work.

Steady-state ion concentration. The steady-state concentration of small ions of either polarity $n_{ss}^{+,-}$ (cm^{-3}) is calculated by balancing ion-pair production (Q), recombination (αn^2), and first-order losses (bn). The pathway-dependent loss frequency $b^{+,-}$ includes contributions from scavenging and from pathway-specific nucleation/consumption terms (defined in sections 3.6 and 3.7). $n_{ss}^{+,-}$ is defined as the following equation. It is set to $n_{ss}^{+,-} = Q/b^{+,-}$ in case equation S6 yields $n_{ss}^{+,-} \lessapprox 1\ cm^{-3}$ to ensure numeric stability of the model.

$$n_{ss}^{+,-}(Q, \alpha, b^{+,-}) = \frac{\sqrt{(b^{+,-})^2 + 4\alpha Q} - b^{+,-}}{2\alpha} \tag{S6}$$

Growth time. The characteristic growth time t_{growth} (s) parameterises the time required for newly formed clusters to grow to a stable cluster size. It is parameterised as:

$$t_{growth}([OOM], [\mathrm{H_2SO_4}]) = \frac{C_t}{[OOM] + [\mathrm{H_2SO_4}]} \tag{S7}$$

where C_t is a fitted scaling factor (Table S2). This form assumes growth is faster when either condensable vapour is abundant, and it neglects explicit temperature dependence in the growth-time parameterisation.

Effective loss frequency during growth. The survival of growing charged clusters depends on an effective first-order loss frequency $1/\tau_{loss}^{+,-}$ (s^{-1}), which combines scavenging (C_s) and neutralisation (αn_{ss}). C_s represents the condensation sink, while α corresponds to the ion-ion recombination coefficient. The specific expression used here arises from substituting eqiation S6 into the combined loss term $C_s + \alpha \cdot n_{ss}$:

$$\frac{1}{\tau_{loss}^{+,-}}(C_s, \alpha, Q, b^{+,-}) = \frac{2C_s - b^{+,-} + \sqrt{(b^{+,-})^2 + 4\alpha Q}}{2} \tag{S8}$$

Survival probability. The survival probability is then calculated assuming first-order loss kinetics over the growth time:

$$P_{surv}^{+,-} = exp(-t_{growth} \cdot (1/\tau_{loss}^{+,-})) \tag{S9}$$

Temperature-dependent prefactors. Several pathway rate coefficients are written in a common exponential form:

$$k(T) = exp(A - B \cdot T) \tag{S10}$$

In the pathway-specific equations below, this form appears as $exp(A - B \cdot T)$; the coefficients A and B are fitted where listed in Table S2 and otherwise fixed by construction.

3.5 Neutral $\mathrm{H_2SO_4}$-based pathways $J_{b,n}$ and $J_{t,n}$

$J_{b,n}$ represents neutral binary nucleation involving $\mathrm{H_2SO_4}$ and water, and $J_{t,n}$ represents neutral ternary nucleation involving $\mathrm{H_2SO_4}$, $\mathrm{NH_3}$, and water. Both pathways follow the fixed CLOUD-based

parameterisation of Dunne *et al.*[19]. These terms are included without refitting in order to (i) preserve compatibility with existing model implementations and (ii) isolate the present fit to OOM-related processes. The complete set of parameters underlying the Dunne *et al.*[19] parameterisation with full numerical precision, is provided in the Gordon *et al.*[20] supplementary information.

3.6 Neutral organic pathways J_0

Neutral OOM-related nucleation is represented as the sum of a purely organic pathway and a mixed OOM-H_2SO_4 pathway:

$$J_0 = J_{0,OOM} + J_{0,mix} \tag{S11}$$

The two components are defined below. The fitted coefficients and exponents are provided in Table S2.

- **Pure OOM neutral pathway $J_{0,OOM}$.** The neutral pure biogenic pathway depends only on the OOM concentration and is defined as:

  $$J_{0,OOM} = exp(A_{0,OOM}) \cdot [OOM]^{\gamma_{0,OOM}} \tag{S12}$$

  The fitted logarithmic prefactor $A_{0,OOM}$ as well as the OOM exponent $\gamma_{0,OOM}$ are listed in Table S2. No statistically significant temperature dependence could be resolved for this pathway.

- **Mixed neutral pathway $J_{0,mix}$.** The mixed neutral pathway represents synergistic nucleation involving both H_2SO_4 and OOMs. It uses a temperature-dependent prefactor together with power-law dependencies on [OOM] and [H_2SO_4]. The prefactor is defined as:

  $$k_{0,mix}(T) = exp(A_{0,mix} - B_{0,mix} \cdot T) \tag{S13}$$

  where $A_{0,mix}$ and $B_{0,mix}$ are fitted coefficients (Table S2). The full mixed neutral nucleation rate is:

  $$J_{0,mix} = k_{0,mix}(T) \cdot [OOM]^{\gamma_{OOM,0,mix}} \cdot [\mathrm{H_2SO_4}]^{\gamma_{SA,0,mix}} \tag{S14}$$

  where $\gamma_{OOM,0,mix}$ and $\gamma_{SA,0,mix}$ are fitted exponents (Table S2).

3.7 Ion-induced pathways J_+ and J_-

We represent ion-induced nucleation using a shared mixed H_2SO_4–OOM initiation term, $J_{Ion,mix}$, which contributes to both the positive and negative ion-induced pathways. This term represents the formation rate of mixed organic–inorganic clusters initiated on charged ions and is combined with polarity-specific ion balances, baseline pathways, and survival probabilities to yield the total positive (J_+) and negative (J_-) ion-induced nucleation rates. This mixed ion component includes (a) a temperature-dependent prefactor, (b) a power-law dependence on the sulfuric acid concentration [H_2SO_4], and (c) a saturating dependence on the OOM concentration [OOM] represented by a Langmuir-type factor.

$$k_{Ion,mix}(T) = exp(A_{Ion,mix} - B_{Ion,mix} \cdot T) \tag{S15}$$

where $A_{Ion,mix}$ and $B_{Ion,mix}$ are fitted coefficients (Table S2). The saturation factor is:

$$f(OOM) = \frac{[OOM]}{K_{OOM}^{Ion,mix} + [OOM]} \tag{S16}$$

where the factor $K_{OOM}^{Ion,mix}$ is set to 1000. The full term is:

$$J_{Ion,mix}(T, [OOM], [H_2SO_4]) = k_{Ion,mix}(T) \cdot f(OOM) \cdot [H_2SO_4]^{\gamma_{Ion,mix}} \quad (S17)$$

where $\gamma_{Ion,mix}$ is the fitted H_2SO_4 exponent (Table S2). The same functional form of $J_{Ion,mix}$ is used for both positive and negative ion-induced nucleation. Differences between J_+ and J_- arise from polarity-specific ion concentrations, loss terms, baseline pathways, and survival probabilities.

3.7.1 Positive ion-induced pathway J_+

We describe positive ion-induced nucleation in an initiation-survival framework. The nucleation rate depends on: (i) the steady-state concentration of positive ions n_{ss}^+ computed from equation S6; (ii) an initiation frequency per ion which includes OOM-only and mixed H_2SO_4–OOM contributions; and (iii) a survival probability P_{surv}^+ (equation S9) accounting for losses during growth. This overall approach is consistent with the ion-balance concept used in Dunne *et al.*[19], but with initiation terms modified to include OOM contributions.

$$J_+ = n_{ss}^+ \cdot (J_{+,OOM} + J_{Ion,mix}) \cdot P_{surv}^+ \quad (S18)$$

- **OOM-only ion initiation component $J_{+,OOM}$.** The temperature-independent prefactor is defined as:

$$k_{+,OOM} = exp(A_{+,OOM}) \quad (S19)$$

  where $A_{+,OOM}$ is a fitted coefficient (Table S2). The OOM-only initiation component is:

$$J_{+,OOM} = k_{+,OOM} \cdot [OOM]^{\gamma_{+,OOM}} \quad (S20)$$

  where $\gamma_{OOM,+,OOM}$ is the fitted OOM exponent (Table S2).

- **Initiation frequency** The initiation frequency per positive ion is defined as the sum: $J_{+,OOM} + J_{Ion,mix}$.

- **Positive ion loss frequency used to compute n_{ss}^+.** The loss term used in equation S6 for positive ions is: $b^+ = C_s + J_{+,OOM} + J_{Ion,mix}$. This choice treats the initiation terms as effective first-order losses of ions in the steady-state balance, consistent with the structure of ion-balance formulations used in CLOUD-based parameterisations.

3.7.2 Negative ion pathway J_-

The negative ion-induced nucleation pathway is the sum of (i) an inorganic negative-ion baseline term inherited from Dunne *et al.*[19] and (ii) a survival-weighted contribution from the mixed H_2SO_4–OOM ion mechanism. This structure reflects the fact that, in the present implementation, the negative-ion inorganic baseline is retained from the Dunne *et al.*[19] parameterisation, while OOM effects enter through the same mixed ion-induced term $J_{Ion,mix}$.

- **Base negative-ion inorganic term $J_{-,SA}$.** The base negative ion-induced nucleation rate involving H_2SO_4–NH_3 is directly inherited from Dunne *et al.*[19] and is defined as:

$$J_{-,SA} = J_{b,i} + J_{t,i} \quad (S21)$$

  The terms $J_{b,i}$ and $J_{t,i}$ each depend on temperature and inorganic precursor concentrations (H_2SO_4 and NH_3)[19]. These quantities are not refitted in the present work and are included to retain the established description of inorganic negative ion-induced nucleation.

- **Mixed H_2SO_4–OOM contribution.** The OOM-related negative-ion contribution uses the same $J_{Ion,mix}$ expression as in the positive ion pathway (equation S17), combined with the negative-ion concentration and survival probability.

- **Negative ion loss frequency and final negative ion-induced nucleation rate.** The negative-ion loss frequency entering equation S6, which determines n_{ss}^{-} and P_{surv}^{-}, is defined as follows

$$b^{-} = k_{b,i}(T) \cdot [\mathrm{H_2SO_4}]^{p_{b,i}} + k_{t,i}(T) \cdot f_i([\mathrm{NH_3}], [\mathrm{H_2SO_4}]) + C_s + J_{Ion,mix} \qquad \text{(S22)}$$

  The functions $k_{b,i}$, $k_{t,i}$ and f_i, as well as the exponent $p_{b,i}$ are defined in Dunne *et al.*[19]. The full negative ion pathway is:

$$J_{-} = J_{-,SA} + J_{Ion,mix} \cdot n_{ss}^{-} \cdot P_{surv}^{-} \qquad \text{(S23)}$$

3.8 Parameter estimation methodology

We estimated the 12 adjustable parameters listed in Table S2 by fitting model predictions (J_{model}) to measurements (J_{meas}). We optimised the parameters by maximising the coefficient of determination (R^2) between base-10 logarithms of measured and modelled nucleation rates to ensure appropriate weighting across orders of magnitude:

$$R_{log}^2 = 1 - \frac{\sum_i (log_{10} J_{meas,i} - log_{10} J_{model,i})^2}{\sum_i (log_{10} J_{meas,i} - \overline{log_{10} J_{meas}})^2} \qquad \text{(S24)}$$

To robustly explore the 12-dimensional parameter space and minimise sensitivity to initial guesses, we used a multi-method optimisation strategy combining deterministic and stochastic algorithms[21;22]. This involved iteratively applying a portfolio of optimisation algorithms (including quasi-Newton, simplex, evolutionary, and non-linear least-squares methods) over a large number ($\sim 10^4$) of sequences initiated from adaptively randomised starting points. We maintained physical constraints, such as positivity for exponents (γ), temperature coefficients (B), and the growth time factor (C_t), during optimisation.

3.9 Simplifications

The dataset underlying the development of this parameterisation is restricted to oxidation products from α-pinene. No other volatile organic precursors were investigated experimentally. In the global model implementation, OOM are represented as oxidation products from bulk monoterpene emissions, of which α-pinene contributes approximately 41 %. The parameterisation therefore assumes that nucleation behaviour derived from α-pinene oxidation products is representative of monoterpene-derived OOM more generally. Application of this parameterisation to environments where other organic precursors (e.g. isoprene) contribute substantially to the condensable vapour pool may introduce additional uncertainties, as their nucleation behaviour was not constrained by the present dataset.

The dependence on OOM concentration within the ion-related $J_{Ion,mix}$ term consistently indicated extremely rapid saturation in initial fits. We implemented this "trigger" behaviour by adopting the Langmuir-isotherm form equation S16 and fixing the saturation constant $K_{Ion,mix}^{OOM} = 1000\ cm^{-3}$.

Fits also consistently yielded negligible temperature coefficients ($B \approx 0$) for the purely organic pathways (neutral $J_{0,OOM}$ and positive ion $J_{+,OOM}$). These pathways were simplified to be T-independent by fixing $B_{0,OOM} = 0$ and $B_{+,OOM} = 0$, as these parameters could not be robustly constrained within the limited temperature range of the dataset.

The use of the same term ($J_{Ion,mix}$, equation S17) contributing to both positive and negative ion channels is a simplification based on the working hypothesis that the initial formation of mixed H_2SO_4–OOM clusters is not strongly polarity dependent. These choices resulted in the final 12-parameter model structure. Together, these simplifications reflect a balance between physical interpretability and the constraints imposed by the available experimental dataset.

3.10 Parameterisation evaluation and interpretation

The individual terms of the parameterisation (Eq. S5) are physically motivated and represent different nucleation pathways, distinguished by their different dependencies on vapour concentrations, ion-pair production rate and temperature. In any given environment, new particles will form from each pathway simultaneously, at rates given by the parameterisation for each term in Eq. S5. The sum of the pathways accounts for the total nucleation rate expected in an environment that combines H_2SO_4, OOM, ions and (only for H_2SO_4 without OOM) NH_3. The relative importance of the individual channels to the total nucleation rate can be determined by evaluating each term separately. So, for example, in an environment with very high sulfuric acid but low OOM, it may turn out that the H_2SO_4-OOM (J_-) term dominates, but nevertheless there are simultaneous contributions from the other pathways—each of which has a physical interpretation, as described above.

Neutral pathways exhibit a strong dependence on OOM concentration, with fitted exponents of $\gamma_{0,OOM} \approx 1.8$ for the purely organic pathway and $\gamma_{OOM,0,mix} \approx 2.1$ for the mixed pathway, indicating an overall approximately quadratic dependence on [OOM]. The mixed neutral pathway further shows a non-linear dependence on sulfuric acid concentration ($\gamma_{SA,0,mix} \approx 0.74$). Ion-induced pathways depend approximately linearly on their dominant precursors, with $J_{+,OOM}$ scaling with [OOM] ($\gamma_{OOM,+,OOM} \approx 1.05$) and the mixed ion pathway scaling with [H_2SO_4] ($\gamma_{Ion,mix} \approx 1.15$).

The influence of OOM concentration on $J_{Ion,mix}$ saturates rapidly due to the fixed low saturation constant $K^{OOM}_{Ion,mix}$, reflecting that very low organic vapour concentrations are sufficient to activate the mixed ion-induced nucleation pathway. As a result, this pathway effectively behaves as an OOM-activated channel: once OOM concentrations exceed $\sim 10^3$ cm^{-3}, this channel's dependence on OOM becomes weaker and the nucleation rate is primarily controlled by H_2SO_4, temperature, and ion-related terms. This behaviour is specific to the ion-induced mixed pathway. In contrast, the neutral pathways ($J_{0,OOM}$ and $J_{0,mix}$) retain a strong dependence on OOM concentration over the full range of conditions considered here, and therefore do not exhibit such early saturation. This distinction reflects the different roles of organic vapours in neutral and charged cluster formation within the parameterisation.

The temperature sensitivity in the fitted part of the model arises primarily from the mixed H_2SO_4–OOM pathways. The neutral pathway $J_{0,mix}$ depends on temperature through the parameter $B_{0,mix}$ and the ion-related pathway $J_{Ion,mix}$ through $B_{Ion,mix}$. The latter exhibits a particularly strong negative temperature dependence ($B_{Ion,mix} \approx 0.173$), which is compensated by a large positive logarithmic prefactor ($A_{Ion,mix} \approx 21.53$) to yield realistic rate magnitudes within the fitted temperature range.

In contrast, the purely organic pathways ($J_{0,OOM}$ and $J_{+,OOM}$) and the cluster growth time parameterisation (t_{growth}) are effectively temperature independent in this simplified representation. As a result, the overall temperature dependence of the total nucleation rate J_{total} is dominated by the mixed organic-inorganic pathways. Because the parameterisation is constrained by measurements at only two temperatures (-10 °C and +5 °C), extrapolation beyond this range should be interpreted with caution.

4 EMAC model configuration

In this work we employ global simulations using the ECHAM/MESSy Atmospheric Chemistry (EMAC) model[23], which integrates the ECHAM5 general circulation model with the MESSy2 framework[24] to represent meteorology, trace gases, and aerosol processes. Simulations are conducted with EMAC (ECHAM5 v5.3.02, MESSy v2.55.2) at T63L90 resolution (∼1.9 ° × 1.9 °, 90 hybrid levels up to ∼80 km) for the full year 2016, using a 7.5-minute time step and hourly output. Large-scale dynamics are nudged toward ERA-Interim reanalysis to maintain realistic atmospheric conditions and transport.

For representation of nucleation and growth in the aerosol phase, we adopted the following submodels: GMXe (aerosol microphysics)[25], NAN (new particle formation)[26], IONS (ion-pair production from cosmic rays and radon decay)[26], ORACLE (Volatility Base Set model for secondary Organic Aerosols)[27]. The gas phase chemistry is simulated by the submodel MECCA[28], which integrates the chemical decomposition mechanism MIM1[29]. Anthropogenic emissions are based on the CEDS inventory[30], while biogenic emissions are calculated online using MEGAN (Model of Emissions of Gases and Aerosols from Nature)[31] based on vegetation type and meteorological conditions.

OOM formation is entirely driven by oxidation of lumped monoterpenes, meaning that other organic species, like isoprene, are not included in the formation of OOM. As α-Pinene accounts for approximately 41 % of the total lumped monoterpene emissions, we adopted the OOM yields for this monoterpene. Specifically, yields of 3.4 % and 0.44 % were used for the reaction with O_3 and OH, respectively[14]. Furthermore, once in the aerosol phase, OOM decompose with a lifetime of 2 hours, based on observational evidence[32].

For this study, we perform two simulations using the same model configuration, differing only in the applied NPF parameterisations: 1) the H_2SO_4–AP-OOM scheme developed in the present study and 2) the H_2SO_4–BioOxOrg scheme[33] in combination with the pure biogenic parameterisation of Kirkby *et al.*[15]. Both simulations include H_2SO_4–NH_3 nucleation calculated according to the parameterisation of Dunne *et al.*[19].

References

[1] M. Graus, M. Müller and A. Hansel, *Journal of the American Society for Mass Spectrometry*, 2011, **21**, 1037–1044.

[2] E. Canaval, N. Hyttinen, B. Schmidbauer, L. Fischer and A. Hansel, *Frontiers in Chemistry*, 2019, **7**, 191.

[3] T. Reinecke, M. Leiminger, A. Jordan, A. Wisthaler and M. Müller, *Analytical Chemistry*, 2023, **95**, 11879–11884.

[4] T. Jokinen, M. Sipilä, H. Junninen, M. Ehn, G. Lönn, J. Hakala, T. Petäjä, R. Mauldin Iii, M. Kulmala and D. Worsnop, *Atmospheric Chemistry and Physics*, 2012, **12**, 4117–4125.

[5] N. Hyttinen, O. Kupiainen-Maatta, M. P. Rissanen, M. Muuronen, M. Ehn and T. Kurtén, *The Journal of Physical Chemistry A*, 2015, **119**, 6339–6345.

[6] A. Kürten, L. Rondo, S. Ehrhart and J. Curtius, *Atmospheric measurement techniques*, 2011, **4**, 437–443.

[7] A. Kürten, L. Rondo, S. Ehrhart and J. Curtius, *The Journal of Physical Chemistry A*, 2012, **116**, 6375–6386.

[8] X.-C. He, J. Shen, S. Iyer, P. Juuti, J. Zhang, M. Koirala, M. M. Kytökari, D. R. Worsnop, M. Rissanen, M. Kulmala, N. M. Maier, J. Mikkilä, M. Sipilä and J. Kangasluoma, *Atmospheric Measurement Techniques*, 2023, **16**, 4461–4487.

[9] M. Heinritzi, M. Simon, G. Steiner, A. C. Wagner, A. Kürten, A. Hansel and J. Curtius, *Atmospheric measurement techniques*, 2016, **9**, 1449–1460.

[10] M. Simon, M. Heinritzi, S. Herzog, M. Leiminger, F. Bianchi, A. Praplan, J. Dommen, J. Curtius and A. Kürten, *Atmospheric measurement techniques*, 2016, **9**, 2135–2145.

[11] J. Curtius, M. Heinritzi, L. J. Beck, M. L. Pöhlker, N. Tripathi, B. E. Krumm, P. Holzbeck, C. M. Nussbaumer, L. Hernández Pardo, T. Klimach, K. Barmpounis, S. T. Andersen, R. Bardakov, B. Bohn, M. A. Cecchini, J.-P. Chaboureau, T. Dauhut, D. Dienhart, R. Dörich, A. Edtbauer, A. Giez, A. Hartmann, B. A. Holanda, P. Joppe, K. Kaiser, T. Keber, H. Klebach, O. O. Krüger, A. Kürten, C. Mallaun, D. Marno, M. Martinez, C. Monteiro, C. Nelson, L. Ort, S. S. Raj, S. Richter, A. Ringsdorf, F. Rocha, M. Simon, S. Sreekumar, A. Tsokankunku, G. R. Unfer, I. D. Valenti, N. Wang, A. Zahn, M. Zauner-Wieczorek, R. I. Albrecht, M. O. Andreae, P. Artaxo, J. N. Crowley, H. Fischer, H. Harder, D. L. Herdies, L. A. T. Machado, C. Pöhlker, U. Pöschl, A. Possner, A. Pozzer, J. Schneider, J. Williams and J. Lelieveld, *Nature*, 2024, **636**, 124–130.

[12] Y. Wang, P. Clusius, C. Yan, K. Dällenbach, R. Yin, M. Wang, X.-C. He, B. Chu, Y. Lu, L. Dada, J. Kangasluoma, P. Rantala, C. Deng, Z. Lin, W. Wang, L. Yao, X. Fan, W. Du, J. Cai, L. Heikkinen, Y. J. Tham, Q. Zha, Z. Ling, H. Junninen, T. Petäjä, M. Ge, Y. Wang, H. He, D. R. Worsnop, V.-M. Kerminen, F. Bianchi, L. Wang, J. Jiang, Y. Liu, M. Boy, M. Ehn, N. M. Donahue and M. Kulmala, *Environmental Science & Technology*, 2021, **56**, 770–778.

[13] M. Simon, L. Dada, M. Heinritzi, W. Scholz, D. Stolzenburg, L. Fischer, A. C. Wagner, A. Kürten, B. Rörup, X.-C. He, J. Almeida, R. Baalbaki, A. Baccarini, P. S. Bauer, L. Beck, A. Bergen, F. Bianchi, S. Bräkling, S. Brilke, L. Caudillo, D. Chen, B. Chu, A. Dias, D. C. Draper, J. Duplissy, I. El-Haddad, H. Finkenzeller, C. Frege, L. Gonzalez-Carracedo, H. Gordon, M. Granzin, J. Hakala, V. Hofbauer, C. R. Hoyle, C. Kim, W. Kong, H. Lamkaddam, C. P. Lee, K. Lehtipalo, M. Leiminger, H. Mai, H. E. Manninen, G. Marie, R. Marten, B. Mentler, U. Molteni, L. Nichman, W. Nie, A. Ojdanic, A. Onnela, E. Partoll, T. Petäjä, J. Pfeifer, M. Philippov, L. L. J. Quélever, A. Ranjithkumar, M. P. Rissanen, S. Schallhart, S. Schobesberger, S. Schuchmann, J. Shen, M. Sipilä, G. Steiner, Y. Stozhkov, C. Tauber, Y. J. Tham, A. R. Tomé, M. Vazquez-Pufleau, A. L. Vogel, R. Wagner, M. Wang, D. S. Wang, Y. Wang, S. K. Weber, Y. Wu, M. Xiao, C. Yan, P. Ye, Q. Ye, M. Zauner-Wieczorek, X. Zhou, U. Baltensperger, J. Dommen, R. C. Flagan, A. Hansel, M. Kulmala, R. Volkamer, P. M. Winkler, D. R. Worsnop, N. M. Donahue, J. Kirkby and J. Curtius, *Atmospheric Chemistry and Physics*, 2020, **20**, 9183–9207.

[14] T. Jokinen, T. Berndt, R. Makkonen, V.-M. Kerminen, H. Junninen, P. Paasonen, F. Stratmann, H. Herrmann, A. B. Guenther, D. R. Worsnop, M. Kulmala, M. Ehn and M. Sipilä, *Proceedings of the National Academy of Sciences*, 2015, **112**, 7123–7128.

[15] J. Kirkby, J. Duplissy, K. Sengupta, C. Frege, H. Gordon, C. Williamson, M. Heinritzi, M. Simon, C. Yan, J. Almeida, J. Tröstl, T. Nieminen, I. K. Ortega, R. Wagner, A. Adamov, A. Amorim, A.-K. Bernhammer, F. Bianchi, M. Breitenlechner, S. Brilke, X. Chen, J. Craven, A. Dias, S. Ehrhart, R. C. Flagan, A. Franchin, C. Fuchs, R. Guida, J. Hakala, C. R. Hoyle, T. Jokinen, H. Junninen, J. Kangasluoma, J. Kim, M. Krapf, A. Kürten, A. Laaksonen, K. Lehtipalo, V. Makhmutov, S. Mathot, U. Molteni, A. Onnela, O. Peräkylä, F. Piel, T. Petäjä, A. P. Praplan,

K. Pringle, A. Rap, N. A. D. Richards, I. Riipinen, M. P. Rissanen, L. Rondo, N. Sarnela, S. Schobesberger, C. E. Scott, J. H. Seinfeld, M. Sipilä, G. Steiner, Y. Stozhkov, F. Stratmann, A. Tomé, A. Virtanen, A. L. Vogel, A. C. Wagner, P. E. Wagner, E. Weingartner, D. Wimmer, P. M. Winkler, P. Ye, X. Zhang, A. Hansel, J. Dommen, N. M. Donahue, D. R. Worsnop, U. Baltensperger, M. Kulmala, K. S. Carslaw and J. Curtius, *Nature*, 2016, **533**, 521–526.

[16] S. Mirme and A. Mirme, *Atmospheric Measurement Techniques*, 2013, **6**, 1061–1071.

[17] D. Stolzenburg, G. Steiner and P. M. Winkler, *Atmospheric Measurement Techniques*, 2017, **10**, 1639–1651.

[18] D. Stolzenburg, L. Fischer, A. L. Vogel, M. Heinritzi, M. Schervish, M. Simon, A. C. Wagner, L. Dada, L. R. Ahonen, A. Amorim, A. Baccarini, P. S. Bauer, B. Baumgartner, A. Bergen, F. Bianchi, M. Breitenlechner, S. Brilke, S. Buenrostro Mazon, D. Chen, A. Dias, D. C. Draper, J. Duplissy, I. El Haddad, H. Finkenzeller, C. Frege, C. Fuchs, O. Garmash, H. Gordon, X. He, J. Helm, V. Hofbauer, C. R. Hoyle, C. Kim, J. Kirkby, J. Kontkanen, A. Kürten, J. Lampilahti, M. Lawler, K. Lehtipalo, M. Leiminger, H. Mai, S. Mathot, B. Mentler, U. Molteni, W. Nie, T. Nieminen, J. B. Nowak, A. Ojdanic, A. Onnela, M. Passananti, T. Petäjä, L. L. J. Quéléver, M. P. Rissanen, N. Sarnela, S. Schallhart, C. Tauber, A. Tomé, R. Wagner, M. Wang, L. Weitz, D. Wimmer, M. Xiao, C. Yan, P. Ye, Q. Zha, U. Baltensperger, J. Curtius, J. Dommen, R. C. Flagan, M. Kulmala, J. N. Smith, D. R. Worsnop, A. Hansel, N. M. Donahue and P. M. Winkler, *Proceedings of the National Academy of Sciences*, 2018, **115**, 9122–9127.

[19] E. M. Dunne, H. Gordon, A. Kürten, J. Almeida, J. Duplissy, C. Williamson, I. K. Ortega, K. J. Pringle, A. Adamov, U. Baltensperger, P. Barmet, F. Benduhn, F. Bianchi, M. Breitenlechner, A. Clarke, J. Curtius, J. Dommen, N. M. Donahue, S. Ehrhart, R. C. Flagan, A. Franchin, R. Guida, J. Hakala, A. Hansel, M. Heinritzi, T. Jokinen, J. Kangasluoma, J. Kirkby, M. Kulmala, A. Kupc, M. J. Lawler, K. Lehtipalo, V. Makhmutov, G. Mann, S. Mathot, J. Merikanto, P. Miettinen, A. Nenes, A. Onnela, A. Rap, C. L. S. Reddington, F. Riccobono, N. A. D. Richards, M. P. Rissanen, L. Rondo, N. Sarnela, S. Schobesberger, K. Sengupta, M. Simon, M. Sipilä, J. N. Smith, Y. Stozkhov, A. Tomé, J. Tröstl, P. E. Wagner, D. Wimmer, P. M. Winkler, D. R. Worsnop and K. S. Carslaw, *Science*, 2016, **354**, 1119–1124.

[20] H. Gordon, J. Kirkby, U. Baltensperger, F. Bianchi, M. Breitenlechner, J. Curtius, A. Dias, J. Dommen, N. M. Donahue, E. M. Dunne, J. Duplissy, S. Ehrhart, R. C. Flagan, C. Frege, C. Fuchs, A. Hansel, C. R. Hoyle, M. Kulmala, A. Kürten, K. Lehtipalo, V. Makhmutov, U. Molteni, M. P. Rissanen, Y. Stozkhov, J. Tröstl, G. Tsagkogeorgas, R. Wagner, C. Williamson, D. Wimmer, P. M. Winkler, C. Yan and K. S. Carslaw, *Journal of Geophysical Research: Atmospheres*, 2017, **122**, 8739–8760.

[21] J. Nocedal, *Numerical optimization*, 2006.

[22] W. H. Press, *Numerical recipes 3rd edition: The art of scientific computing*, Cambridge university press, 2007.

[23] E. Roeckner, R. Brokopf, M. Esch, M. Giorgetta, S. Hagemann, L. Kornblueh, E. Manzini, U. Schlese and U. Schulzweida, *Journal of Climate*, 2006, **19**, 3771–3791.

[24] P. Jöckel, A. Kerkweg, A. Pozzer, R. Sander, H. Tost, H. Riede, A. Baumgaertner, S. Gromov and B. Kern, *Geoscientific Model Development*, 2010, **3**, 717–752.

[25] K. Pringle, H. Tost, S. Message, B. Steil, D. Giannadaki, A. Nenes, C. Fountoukis, P. Stier, E. Vignati and J. Lelieveld, *Geoscientific Model Development*, 2010, **3**, 391–412.

[26] S. Ehrhart, E. M. Dunne, H. E. Manninen, T. Nieminen, J. Lelieveld and A. Pozzer, *Geoscientific Model Development*, 2018, **11**, 4987–5001.

[27] A. P. Tsimpidi, V. A. Karydis, A. Pozzer, S. N. Pandis and J. Lelieveld, *Geoscientific Model Development*, 2018, **11**, 3369–3389.

[28] R. Sander, A. Baumgaertner, D. Cabrera-Perez, F. Frank, S. Gromov, J.-U. Grooß, H. Harder, V. Huijnen, P. Jöckel, V. A. Karydis, K. E. Niemeyer, A. Pozzer, H. Riede, M. G. Schultz, D. Taraborrelli and S. Tauer, *Geoscientific model development*, 2019, **12**, 1365–1385.

[29] P. Jöckel, H. Tost, A. Pozzer, C. Brühl, J. Buchholz, L. Ganzeveld, P. Hoor, A. Kerkweg, M. G. Lawrence, R. Sander, B. Steil, G. Stiller, M. Tanarhte, D. Taraborrelli, J. van Aardenne and J. Lelieveld, *Atmospheric Chemistry and Physics*, 2006, **6**, 5067–5104.

[30] E. E. McDuffie, S. J. Smith, P. O'Rourke, K. Tibrewal, C. Venkataraman, E. A. Marais, B. Zheng, M. Crippa, M. Brauer and R. V. Martin, *Earth System Science Data Discussions*, 2020, **2020**, 1–49.

[31] A. Guenther, X. Jiang, C. L. Heald, T. Sakulyanontvittaya, T. a. Duhl, L. Emmons and X. Wang, *Geoscientific Model Development*, 2012, **5**, 1471–1492.

[32] V. Pospisilova, F. D. Lopez-Hilfiker, D. M. Bell, I. El Haddad, C. Mohr, W. Huang, L. Heikkinen, M. Xiao, J. Dommen, A. S. H. Prevot, U. Baltensperger and J. G. Slowik, *Science Advances*, 2020, **6**, eaax8922.

[33] F. Riccobono, S. Schobesberger, C. E. Scott, J. Dommen, I. K. Ortega, L. Rondo, J. Almeida, A. Amorim, F. Bianchi, M. Breitenlechner, A. David, A. Downard, E. M. Dunne, J. Duplissy, S. Ehrhart, R. C. Flagan, A. Franchin, A. Hansel, H. Junninen, M. Kajos, H. Keskinen, A. Kupc, A. Kürten, A. N. Kvashin, A. Laaksonen, K. Lehtipalo, V. Makhmutov, S. Mathot, T. Nieminen, A. Onnela, T. Petäjä, A. P. Praplan, F. D. Santos, S. Schallhart, J. H. Seinfeld, M. Sipilä, D. V. Spracklen, Y. Stozhkov, F. Stratmann, A. Tomé, G. Tsagkogeorgas, P. Vaattovaara, Y. Viisanen, A. Vrtala, P. E. Wagner, E. Weingartner, H. Wex, D. Wimmer, K. S. Carslaw, J. Curtius, N. M. Donahue, J. Kirkby, M. Kulmala, D. R. Worsnop and U. Baltensperger, *Science*, 2014, **344**, 717–721.
