# Supplementary figures and images for: Nucleation of α-pinene oxidation products with sulfuric acid
Source: Environ Sci Atmos. 2026 Jun 9. Online ahead of print. doi: 10.1039/d6ea00046k (PMC13285975; doi:10.1039/d6ea00046k)

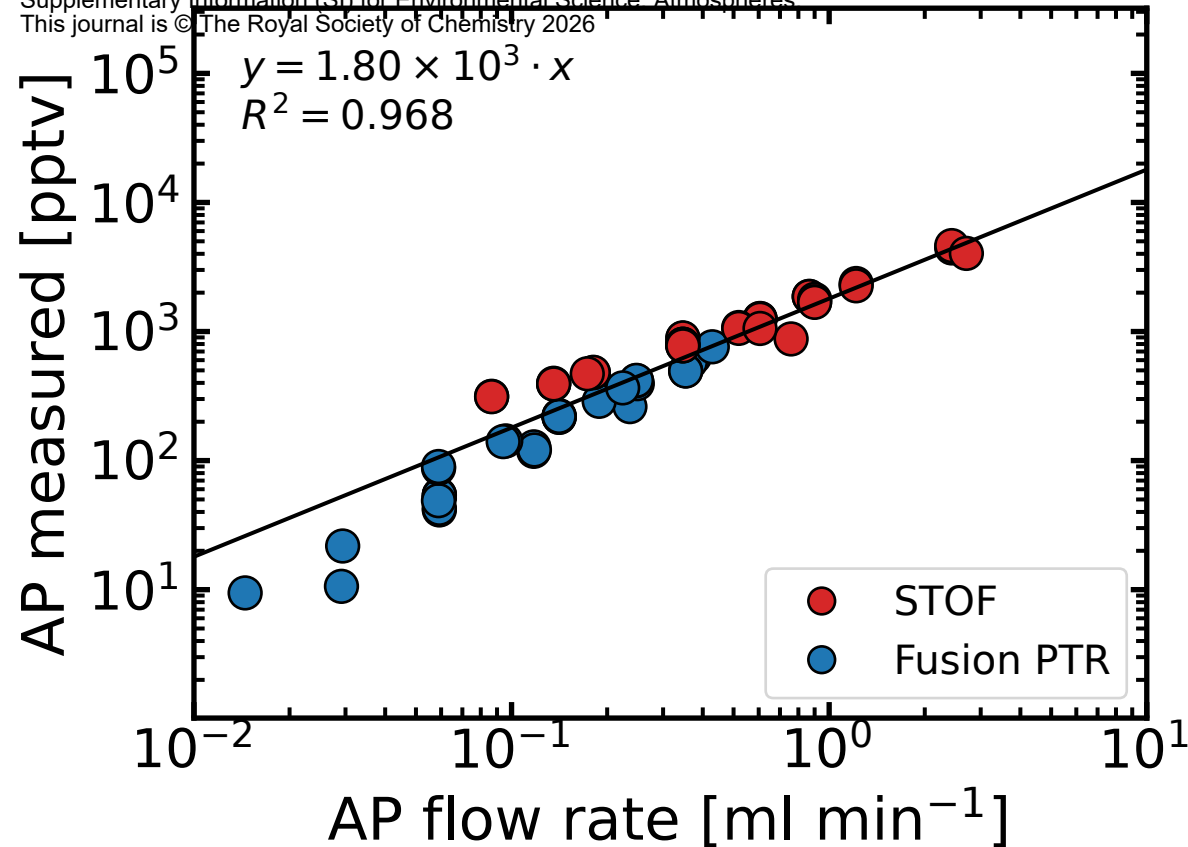

Supplement: EA-OLF-D6EA00046K-s002 [file EA-OLF-D6EA00046K-s002.pdf]

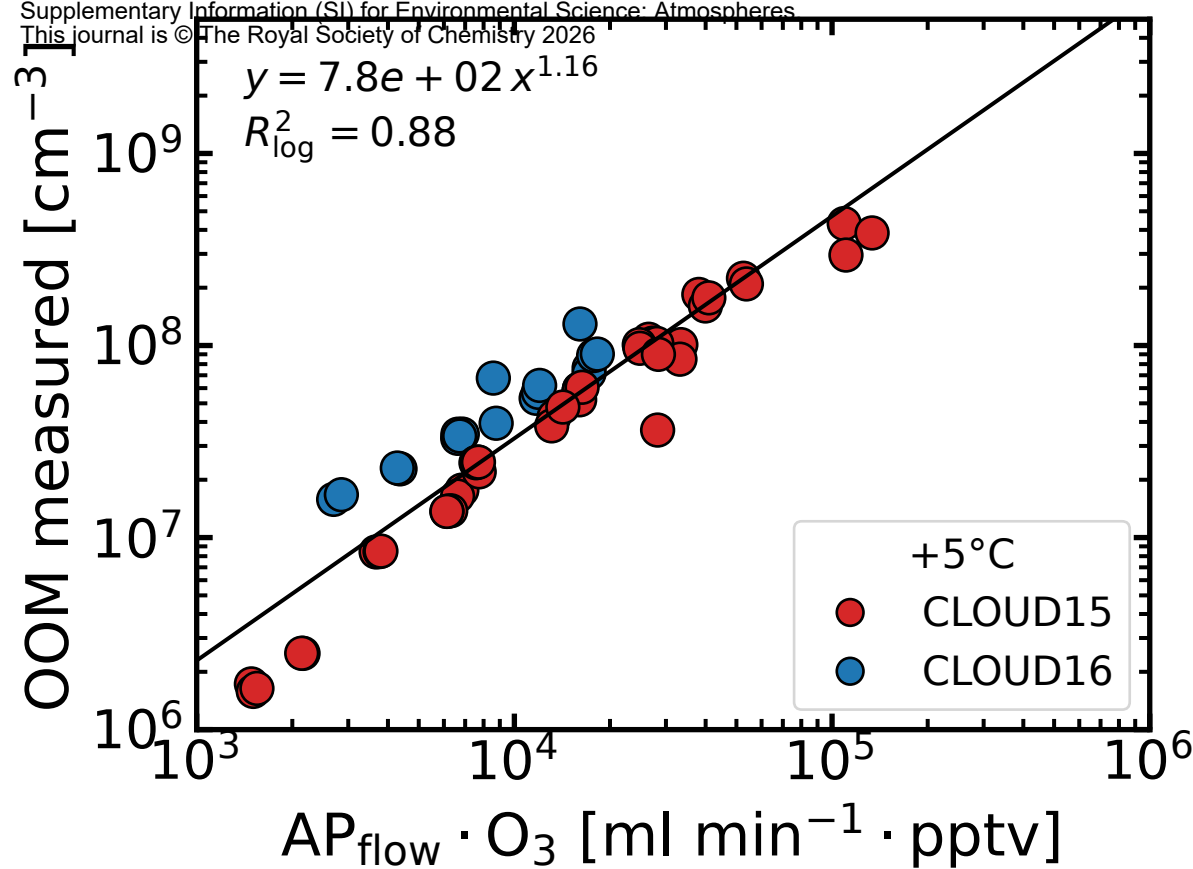

Supplement: EA-OLF-D6EA00046K-s003 [file EA-OLF-D6EA00046K-s003.pdf]

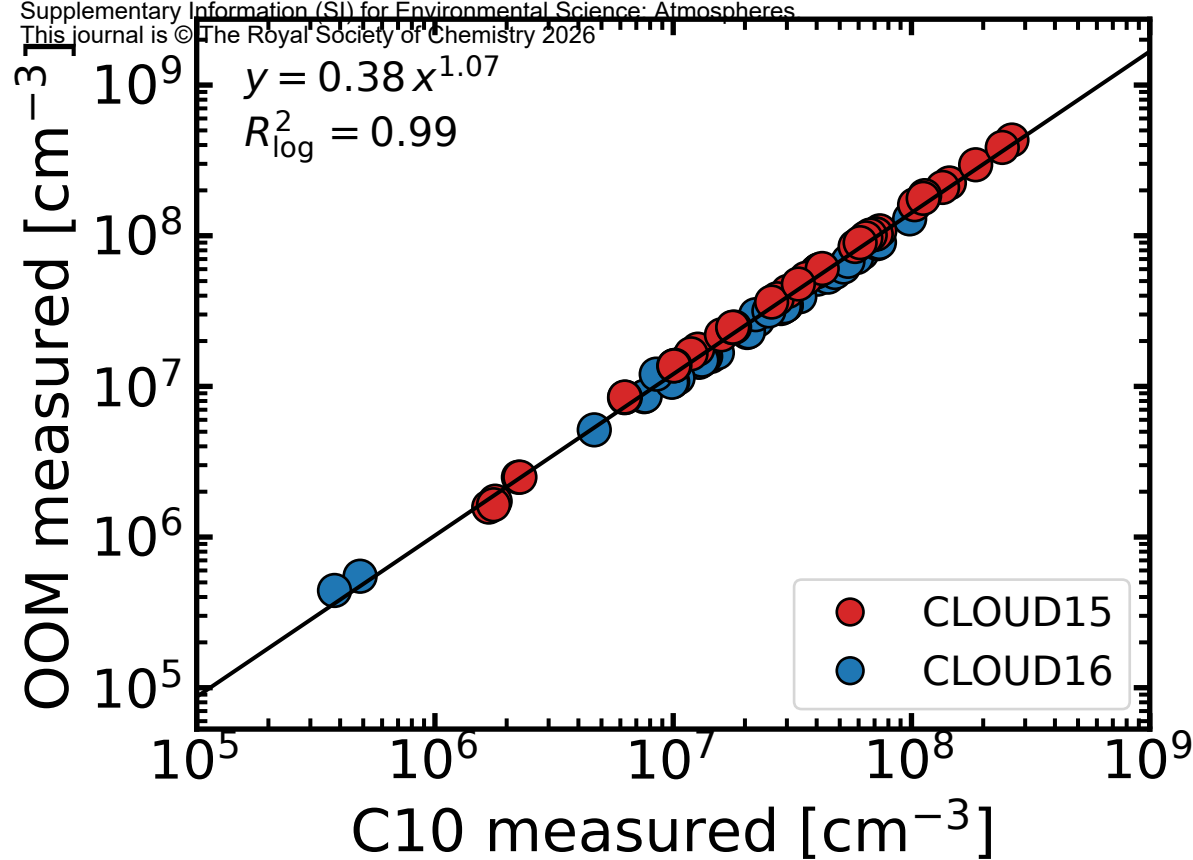

Supplement: EA-OLF-D6EA00046K-s004 [file EA-OLF-D6EA00046K-s004.pdf]

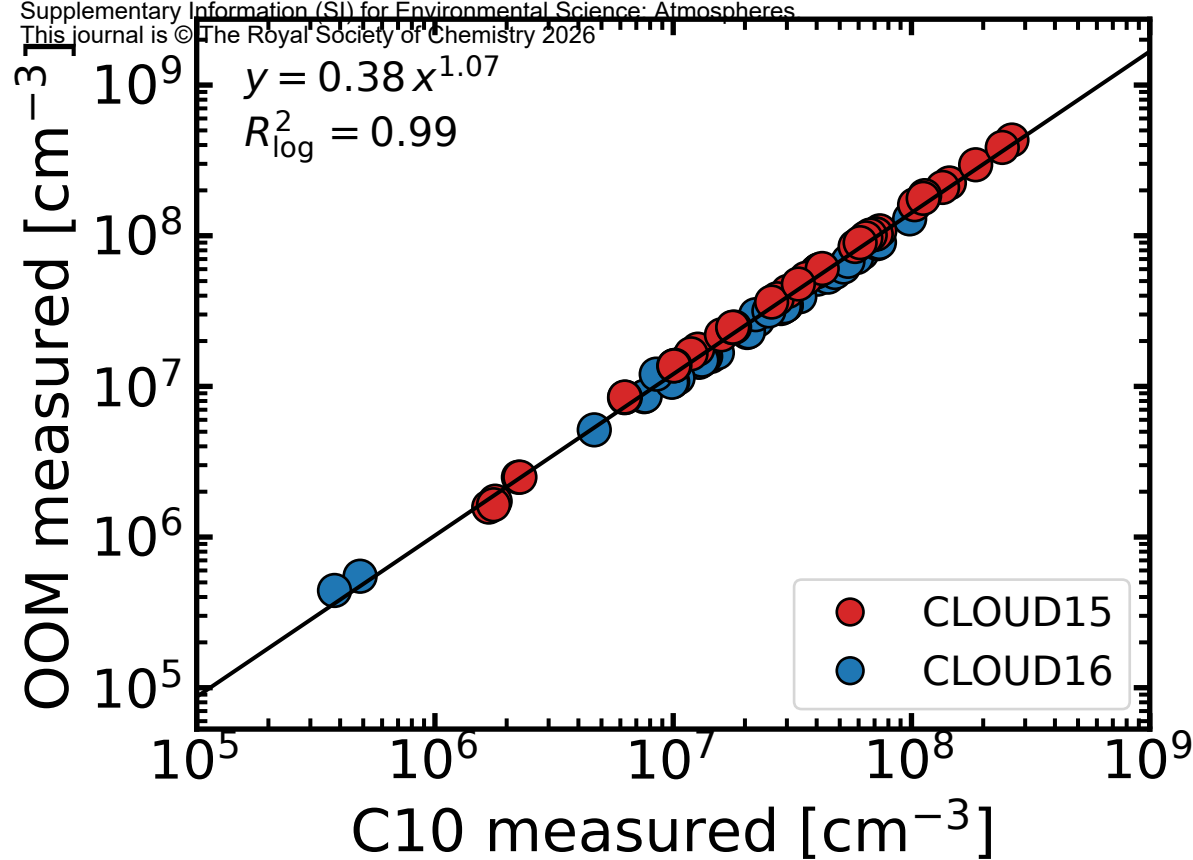

Supplement: EA-OLF-D6EA00046K-s005 [file EA-OLF-D6EA00046K-s005.pdf]

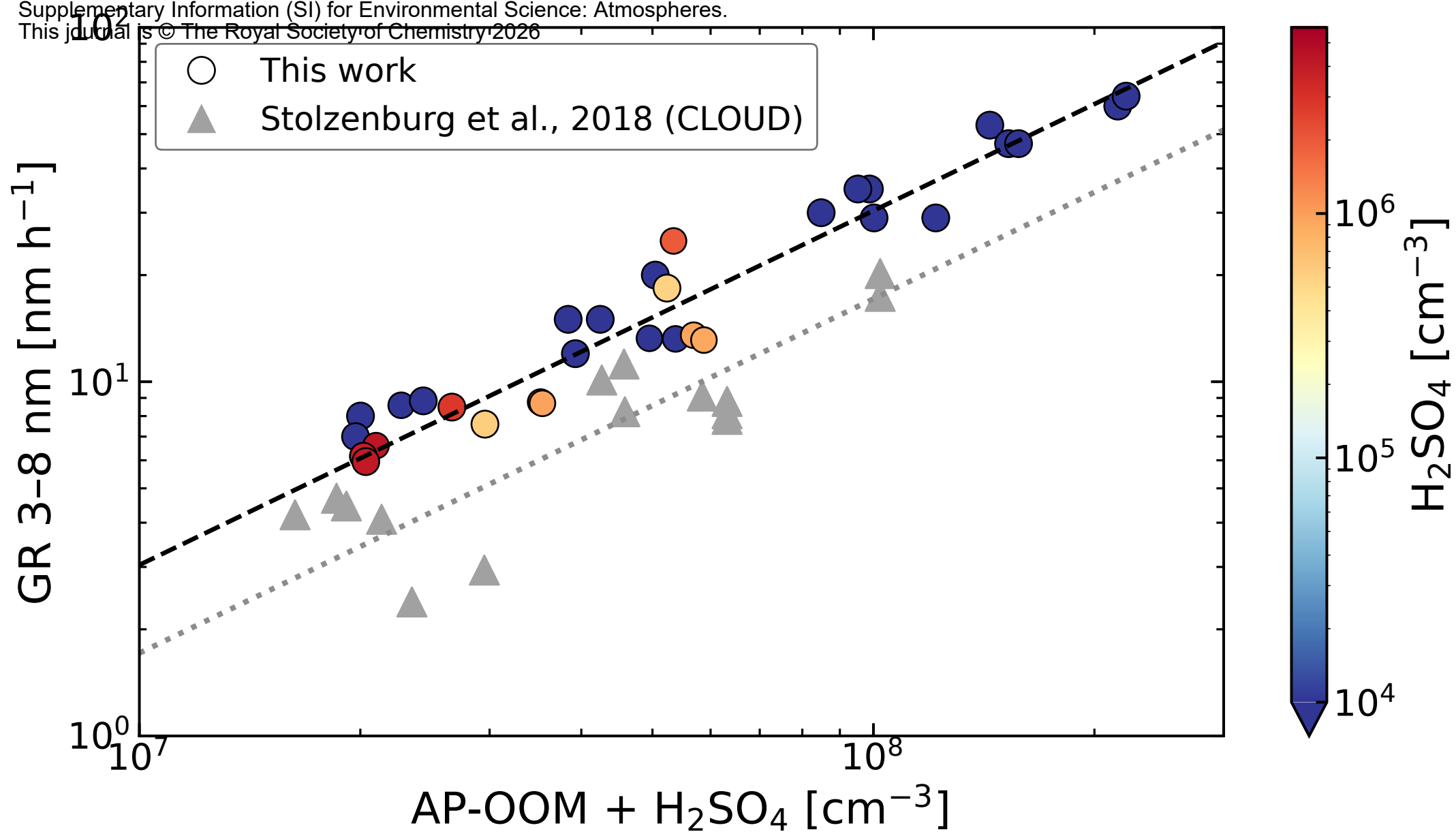

Supplement: EA-OLF-D6EA00046K-s006 [file EA-OLF-D6EA00046K-s006.pdf]

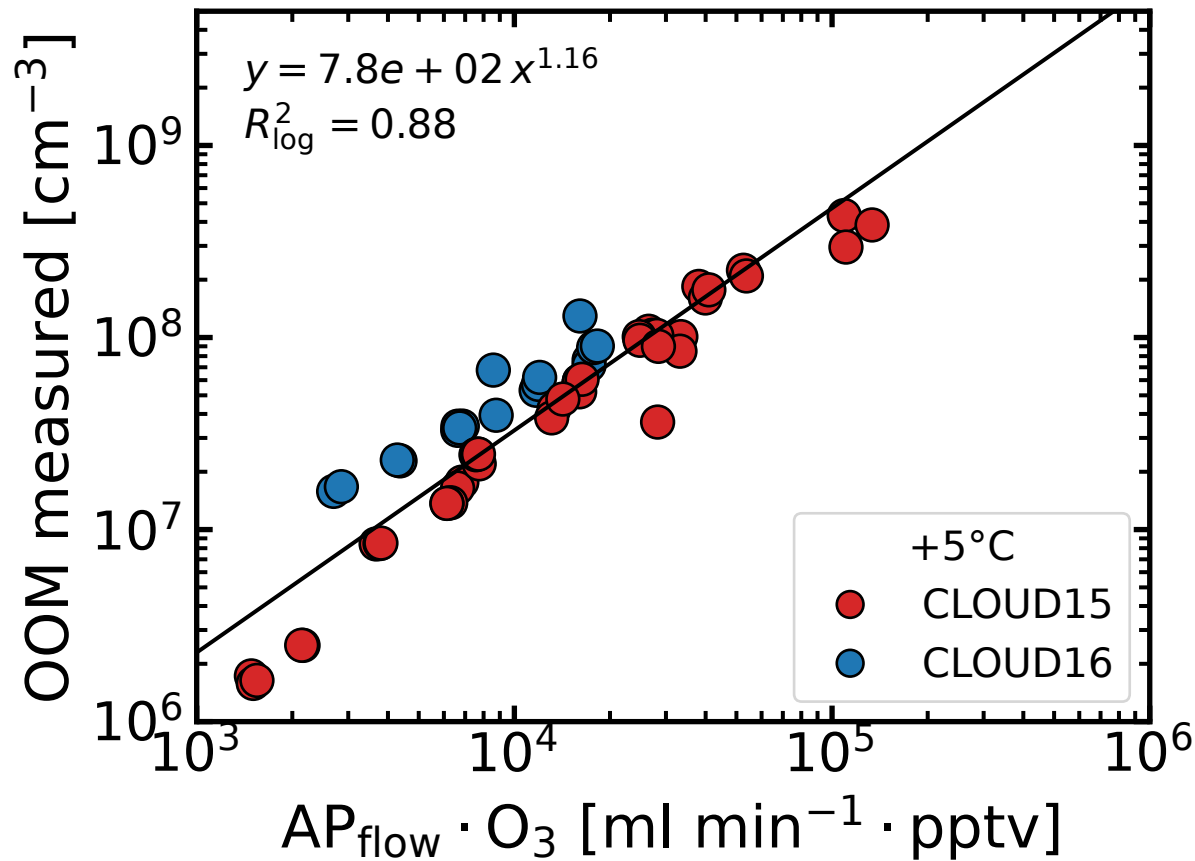

Supplement: EA-OLF-D6EA00046K-s007 [file EA-OLF-D6EA00046K-s007.zip › SI Figures/SI_fig_APcalc_O3_vs_OOM.pdf]

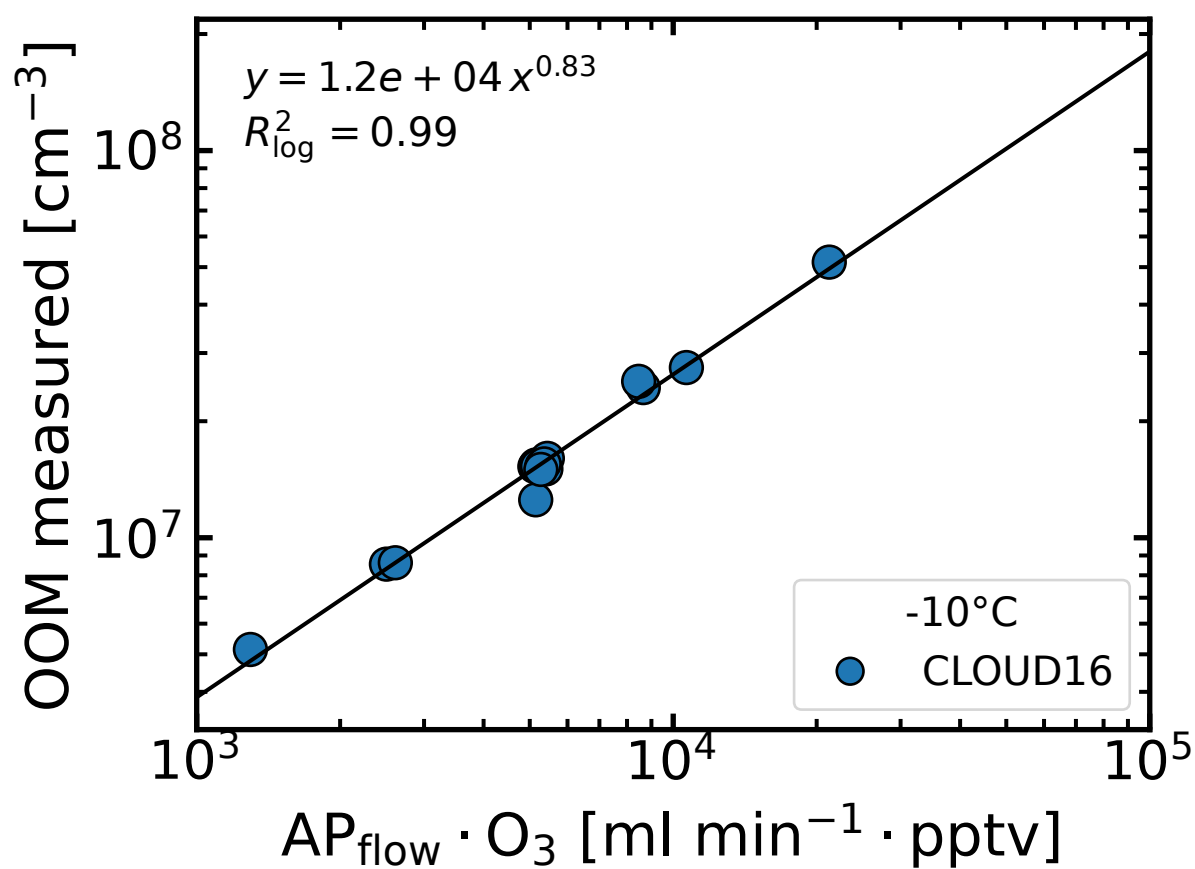

Supplement: EA-OLF-D6EA00046K-s007 [file EA-OLF-D6EA00046K-s007.zip › SI Figures/SI_fig_APcalc_O3_vs_OOM_min10.pdf]

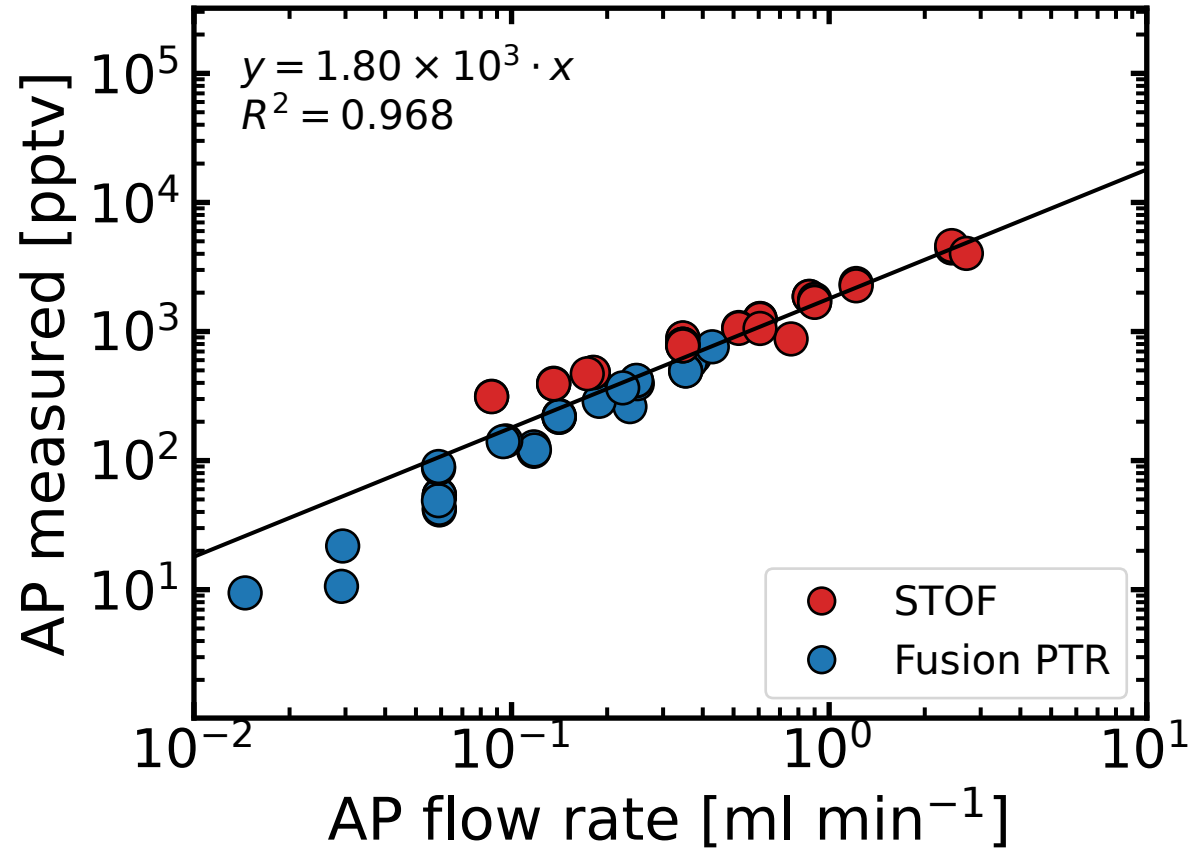

Supplement: EA-OLF-D6EA00046K-s007 [file EA-OLF-D6EA00046K-s007.zip › SI Figures/SI_fig_APmfc_vs_APmeas.pdf]

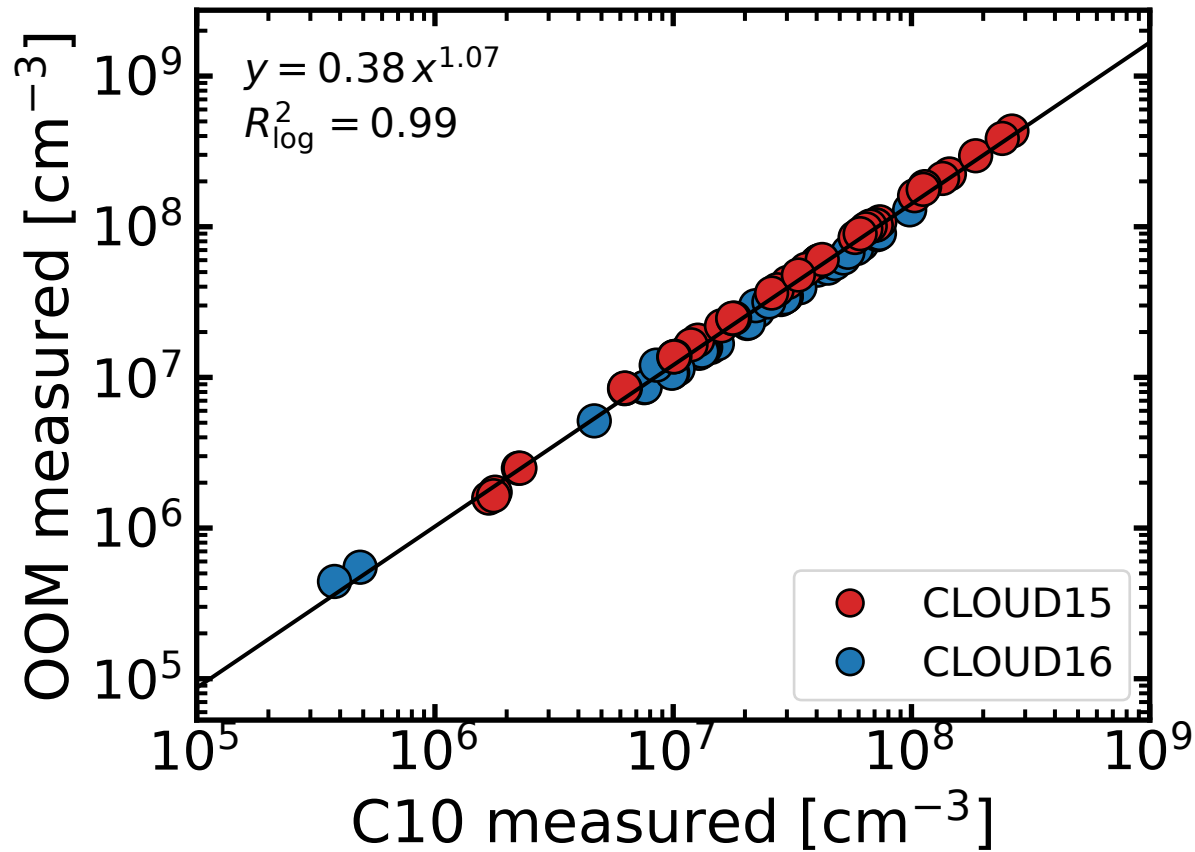

Supplement: EA-OLF-D6EA00046K-s007 [file EA-OLF-D6EA00046K-s007.zip › SI Figures/SI_fig_OOM_vs_C10.pdf]

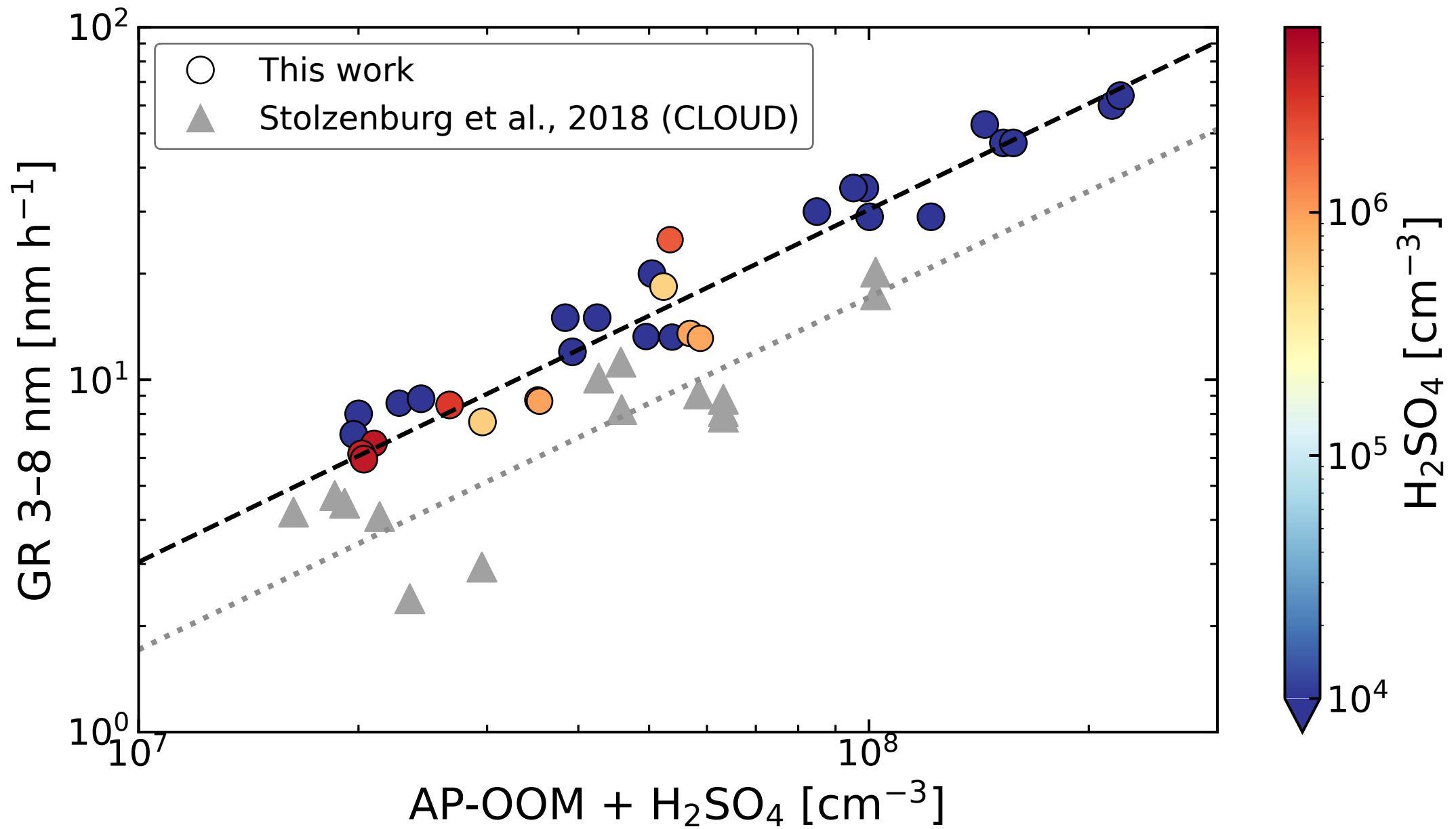

Supplement: EA-OLF-D6EA00046K-s007 [file EA-OLF-D6EA00046K-s007.zip › SI Figures/SI_GRs_forced_origin.pdf]
